# Supplementary material for: Crystal structures of AztD provide mechanistic insights into direct zinc transfer between proteins
Source: Commun Biol. 2019 Aug 9;2:308. doi: 10.1038/s42003-019-0542-z (PMC6689063; doi:10.1038/s42003-019-0542-z)
Supplement: Supplementary file 1 — Supplementary_Information [file 42003_2019_542_MOESM1_ESM.pdf]

SUPPLEMENTARY FIGURES

```
Pden    1 MLRHLAGASALALTLAG--AG----------------FA|QDHDHDHEDVTLYRVFVGDHE
Ckos    1 MMENIMKKRLLSTSISTLLLG----------------LSVMPAFA|DEDVTAWRLFIADHD
Dmur    1 MQKLLLSALTAALALVP--A------------------------AGA|ETTATRLVVADAK
Cmas    1 MKRTAAFAALLSTGLTLCACTNPTSHTA|RTAHTGPAELAELTELAGVSSLHPRL-TYTHD

                                                                   #
Pden   43 KGQVTAFDLAEPDHRWTFPTTGQVK-LYSVAGGAVVAAVQSDADTVQFIRSGISFHDHGD
Ckos   45 KPVVNVIDALDGDKLATFNVKGPAN-LSRSESGATIFAIQGSAGVVSTIASGIAFHDHGD
Dmur   35 THVLSVVDLAQGQTLARFGTPGKLSGLYTGPGGTLAYALHRDDHRVTVLHSGLETVDHGD
Cmas   60 G-GITTLDAVTGEPLATTAREGFLR-LNPTGDGRTLLVTAGDEFL--LYDAAARRVDHGD
                                                             *     *

          # #                   #
Pden  102 HRDIEVGDPAAIDASLTGPRPFHLVEHDGKVVLNYDQGGYAEILDGHALAEGKAEP-GRF
Ckos  104 HADIDIDAPKLLPLELTGKKPGHFVERQGKIAQWFDGEDSAQILGESAVLKGQKNI-TKV
Dmur   95 HRDLVEKPPHVLATLNVGRQPTHFFARDERVAIFNDASGDVAVFGEA-LLGKTNDM-QVV
Cmas  116 HSHAYSGDPVLTSTAFDAPRAGHVVAHGGTTALFSDGEGSATLLPTDSFTQASPAPARRV
          * *                 * *            *

                #                                                    #
Pden  161 PQARAHHGFVAPLGGNWLSTVASDEKVEGDASVPRLGLQAFDAEGNPA--GNLATCTGIH
Ckos  163 NVVAPHHGVAVPYDNYAVVSIPNPDD----ASKRPVGARVVDLQGKKV--GDDALCPGLH
Dmur  153 KVAQPDHGAPALIGDVLLSGYLRLGRV-----------DAYDVRTGRLLQTLDAPCPALH
Cmas  176 ATGSAHHGVAVPLGAGLLATAGTEEE----R----STVRLLDAQGHAS--AETTDCPGVH
                **                                               *   *

Pden  219 GEAFSGAYLAAGCKEGVLTVKAGANGSEYKLLPYPADLPQGVTTGTLLGSTGIQVFLGNY
Ckos  217 GSAGSGDTFALSCETGLLLITQKNAAPVIRHLPYAKTLPEG-STSTLIGGKGMQYFIGNY
Dmur  202 GEAVRGQTAYFGCADGVLAVTVQAQQLRARKLGHPAQTPPSTRVGTVAAHEKSTVVYGNF
Cmas  226 GEAVAGERVVFGCENGPVVFDGTGFAKVPVDTAYQ-------RSGNLAGHPDSPYVLGDH
          *           *  *

Pden  279 G---------PDGLVVIDPVDEPHYRYIKLPFRRVDFALDPAKPSTGYVLTEDGSLHRID
Ckos  276 G---------PDRIILVDPTESDSFRLIQLPTRRVHFVVDPVRAKFAYVFTEDGKLNQID
Dmur  262 G---------N-GLARWTAQD-AALTPVALPAAPLKFTFTA-DGQRLVVLTADGGLHLLH
Cmas  279 KTDPHAEHERPTAVAIIDARD-ASLRTVDLGASYWFRSLGRDESGRGIVLTTDGALKIID
                                                              **

Pden  330 LLKAEIVASAKVTEPYSMDGHWNDPRPRIAMAGDEIVVTDPNAGLVRRIATEDLSERGTV
Ckos  327 VLKGEISQSVRVTDPYSMDGHWNDPRPRIAVADNKIYVTDPLKSKIIVLDATSFKKTSEI
Dmur  310 APTGRLLRSAPVVPPTDA-ADKAAVRPALAVGADHAYVSSPTAGEVVEVSLTDLKVTRRL
Cmas  338 MERGEVLRSIPVIEPWEEKEDWQEPGPILKVAGSTAYVSDATSGLV-VVDLEAGEAVARH
                                                  *

                            #
Pden  390 PVEGKPYNIAVTGGSGVTH----------------
Ckos  387 SVEGQPFNIVAVGGSGKVHGEHHDHEAHHHDDHAH
Dmur  369 AVGGTPAHLTLTSAAGERH----------------
Cmas  397 EVEHAPVEMEVTGV---------------------
               *
```

Supplementary Figure 1. Multiple sequence alignment of AztD homologues from different bacterial phyla using T-Coffee [30]. Identical residues are shaded in black and similar residues in gray. Predicted signal sequence cleavage sites (|) were determined by SignalP 4.0 [31]. Zinc ligands (#) are indicated above the sequences and residues conserved in >95% (*) of all AztD sequences are shown below. The residue indicated by (*) is conserved as either Asp or His in >95% of all sequences. Species abbreviations are as follows: *Paracoccus denitrificans* (Pden), *Citrobacter koseri* (Ckos), *Deinococcus murrayi* (Dmur), *Corynebacterium mastitidis* (Cmas).

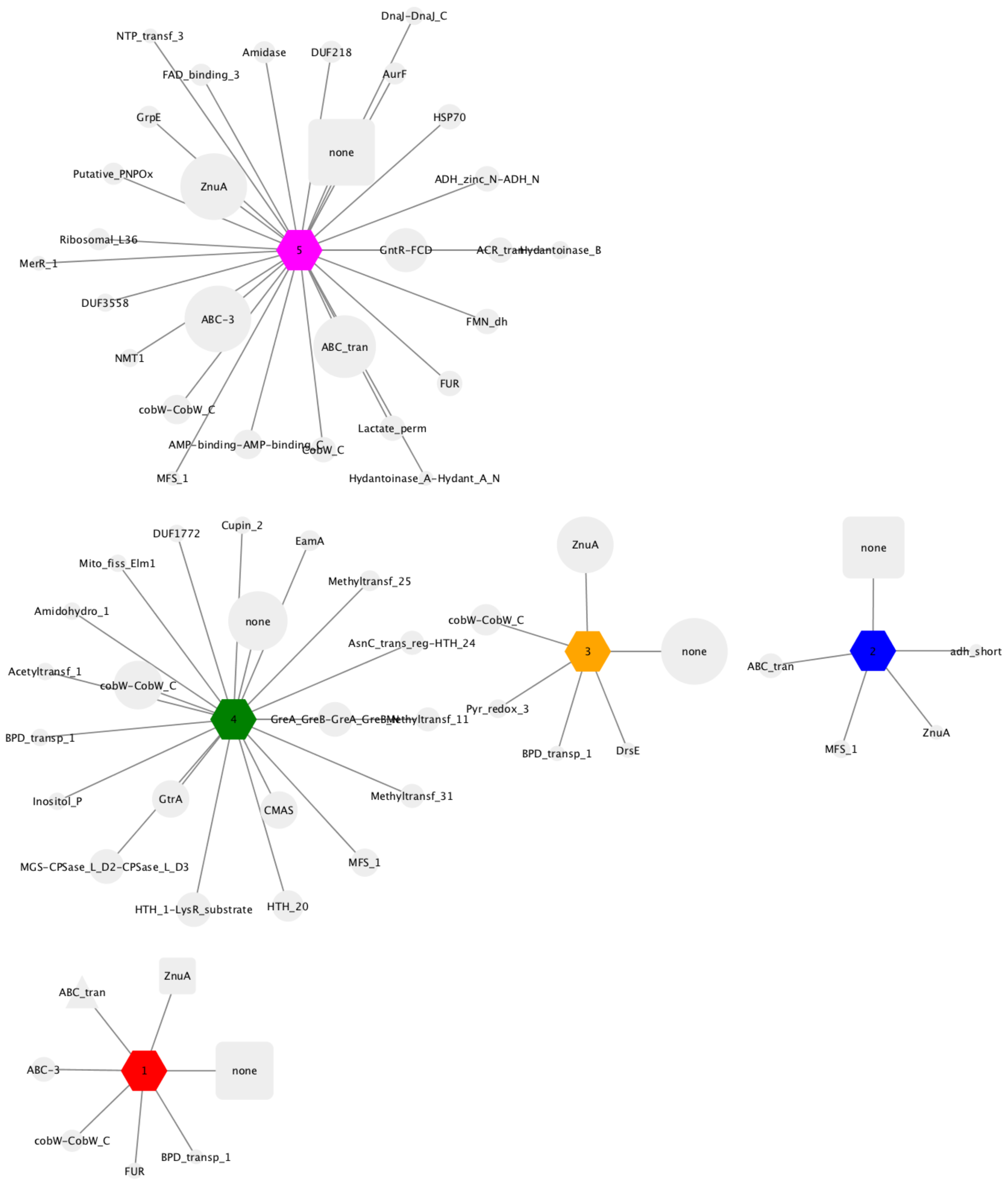

Supplementary Figure 2. Genome neighborhood networks where the central hub represents clusters indicated in Figure 1A.

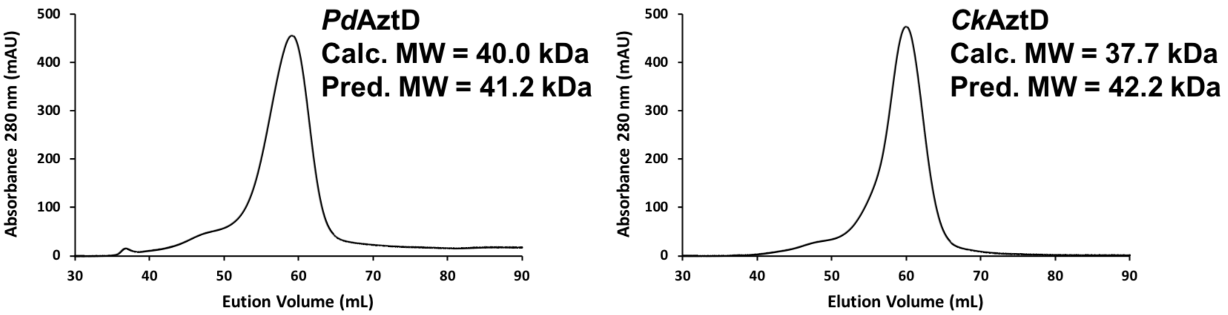

Supplementary Figure 3. Size exclusion chromatograms collected during final purification of *Pd*AztD (left) and *Ck*AztD (right). Calculated MW refers to the MW as determined by elution volume compared to a series of known standards. Predicted MW refers to the expected mass for the processed protein lacking the N-terminal periplasmic targeting sequence. Proteins eluting at higher MW were identified as contaminants by SDS-PAGE.

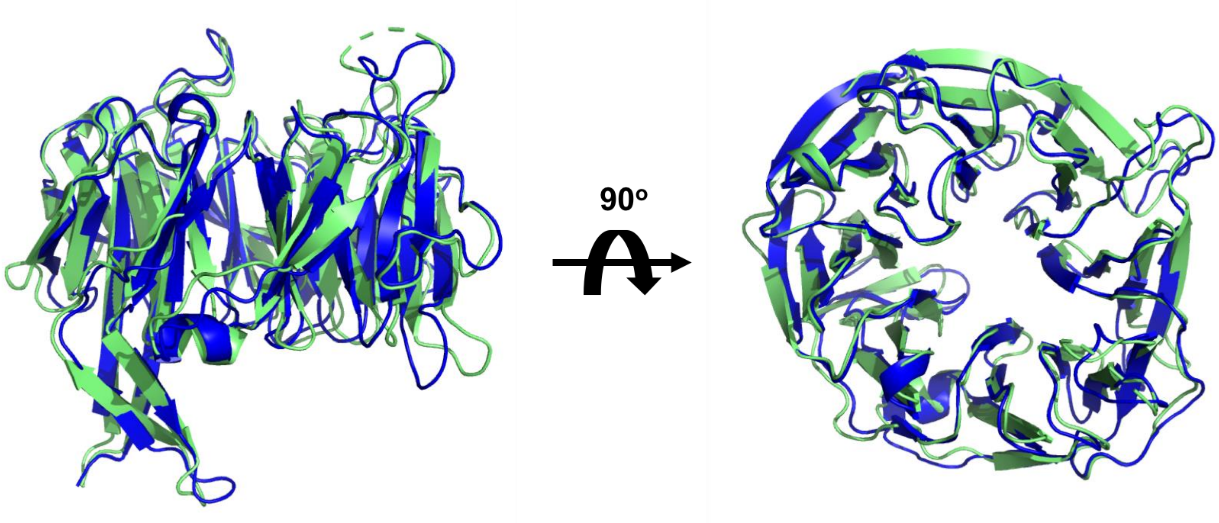

Supplementary Figure 4. Alignment of *Pd*AztD (green) and *Ck*AztD (blue).

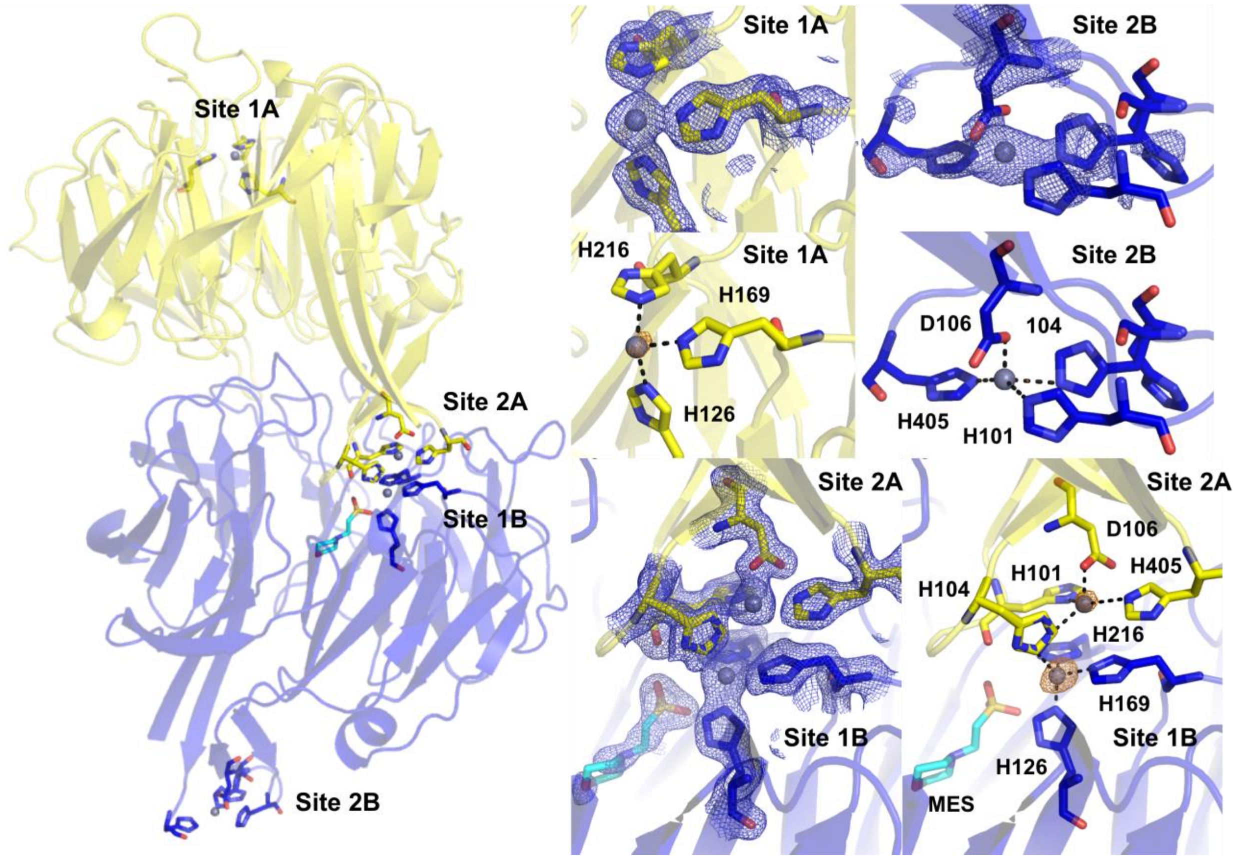

Supplementary Figure 5. Asymmetric unit of the *Ck*AztD crystal structure colored by chain showing each zinc site. 2Fo-Fc (blue mesh) is contoured to 1.0 σ and anomalous difference density (orange mesh) is contoured to 5.0 σ.

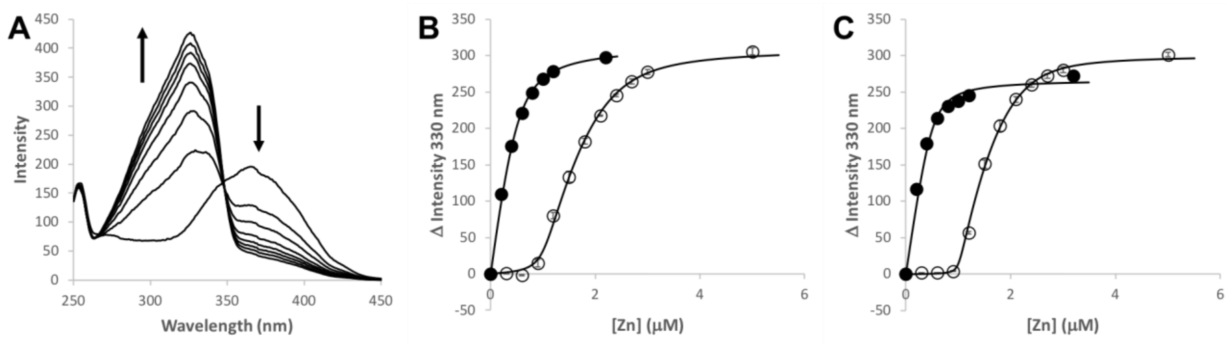

Supplementary Figure 6. Zinc binding by ΔS1 *Pd*AztD and ΔS2 *Pd*AztD by MF-2 competition assay. (A) Example of a titration of 0.5 μM Mag-Fura 2 with zinc. Arrows indicate the direction of fluorescence changes with increasing zinc. Intensity change at 330 nm with increasing zinc in the absence (solid circles) and presence (empty circles) of (B) ΔS1 *Pd*AztD and (C) ΔS2 *Pd*AztD. Titrations containing protein were performed in triplicate (n=3) and error bars represent the standard error between experiments. Fits are shown as solid lines.

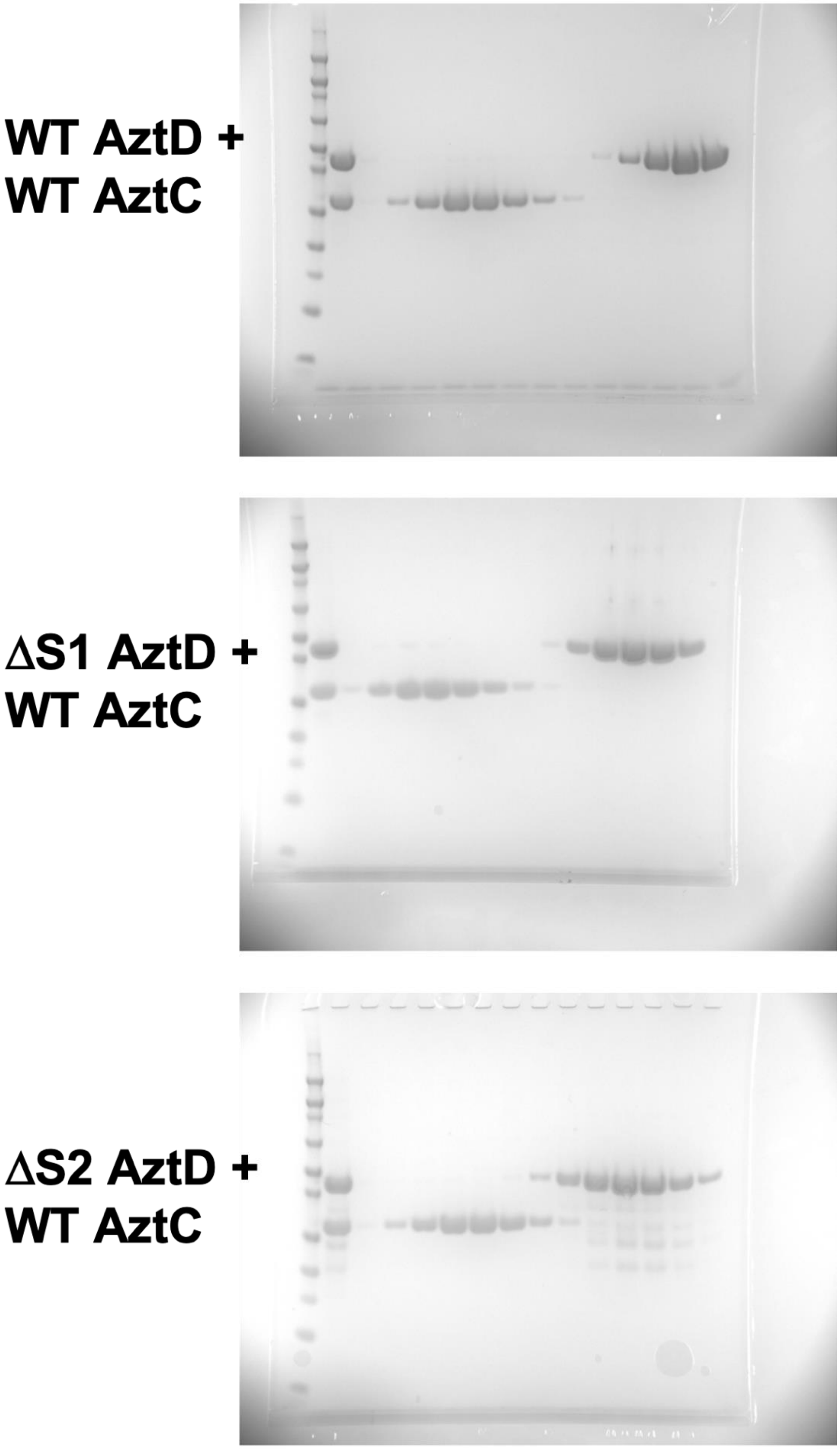

Supplementary Figure 7. Uncropped gel images of chromatographic fractions of WT and mutant AztD reconstituted with zinc and incubated with WT apo AztC (See main text Figure 5).

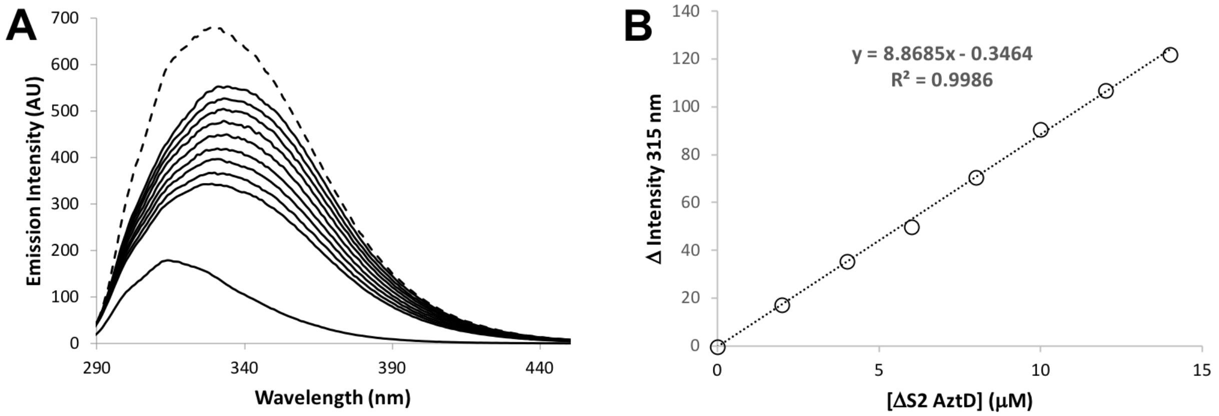

Supplementary Figure 8. Intrinsic fluorescence of 10 μM apo *Pd*AztC titrated with reconstituted ΔS2 *Pd*AztD in the presence of 1 mM EDTA and 10 μM apo ΔS1 *Pd*AztD. Fluorescence emission spectra (A) were recorded after addition of 10 μM apo ΔS1 *Pd*AztD and after each addition of reconstituted ΔS2 *Pd*AztD. The intensity change at 315 nm plotted as a function of reconstituted ΔS2 *Pd*AztD concentration (B). The formula for linear fit is indicated in B. A saturating concentration of $ZnCl_2$ was added after the titration to assess whether transfer from AztD was complete (dotted line, A).

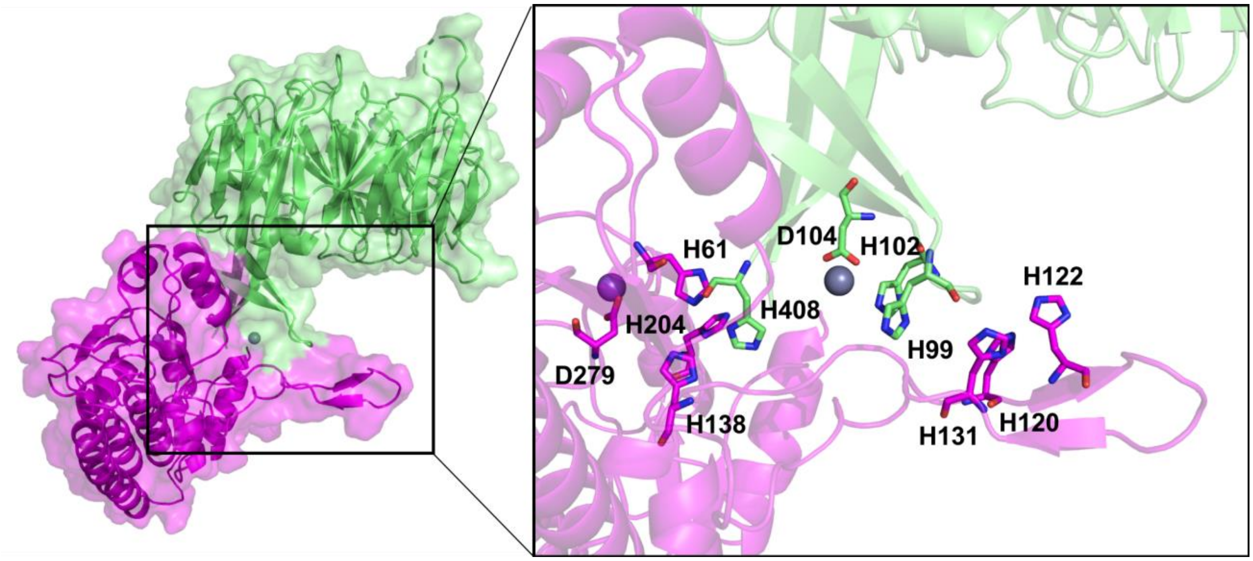

Supplementary Figure 9. Docking model starting from isolated structures of apo *Pd*AztC (magenta) and *Pd*AztD (green). The zoomed in region shows relevant residues in stick form colored according to element. Zinc is shown as a gray sphere and sodium shown as a purple sphere.

SUPPLEMENTARY TABLES

SUPPLEMENTARY TABLE 1

Distribution of species encoding *aztD* genes among bacterial taxa. [a]Phyla are indicated in bold and classes are indicated in italics.

Taxon[a]	Number of genomes with *aztD* Genes
**Proteobacteria**	394
Alphaproteobacteria	334
Gammaproteobacteria	52
Deltaproteobacteria	7
Oligoflexia	1
**Actinobacteria**	146
**Deinococcus-Thermus**	20
**Chloroflexi**	2
**Bacteroidetes**	4
**N/A (Metagenomics)**	11

SUPPLEMENTARY TABLE 2

Ligand to zinc distances for *Pd*AztD

Chain	Site	Ligand	Distance (Å)
A	1	His124 Nε	2.1
		His167 Nε	2.1
		His218 Nε	2.7
		H_2O	2.5
	2	His99 Nε	nd
		His102 Nε	1.9
		Asp104 Oδ1	2.2
		His408 Nε	2.0
B	1	His124 Nε	2.2
		His167 Nε	2.0
		His218 Nε	2.3
		H_2O	2.5
	2	His99 Nε	3.5
		His102 Nε	3.0
		Asp104 Oδ1	2.4
		His408 Nε	2.5
C	1	His124 Nε	2.2
		His167 Nε	2.1
		His218 Nε	2.5
		H_2O	2.7
	2	His99 Nε	nd
		His102 Nε	nd
		Asp104 Oδ1	2.3
		His408 Nε	2.9
D	1	His124 Nε	2.2
		His167 Nε	2.0
		His218 Nε	2.3
		H_2O	2.8
	2	His99 Nε	2.0
		His102 Nε	2.2
		Asp104 Oδ1	2.0
		His408 Nε	2.0

SUPPLEMENTARY TABLE 3

Ligand to zinc distances for *Ck*AztD

Chain	Site	Ligand	Distance (Å)
A	1	His126 Nε	2.4
		His169 Nε	2.2
		His216 Nε	2.4
		H_2O	nd
	2	His101 Nδ	2.6
		His104 Nε	2.5
		Asp106 Oδ1	2.2
		His405 Nε	2.9
B	1	His126 Nε	2.0
		His169 Nε	2.2
		His216 Nε	2.0
		A His104 Nε	2.4
	2	His101 Nδ	2.8
		His104 Nε	2.8
		Asp106 Oδ1	2.2
		His405 Nε	2.8

SUPPLEMENTARY TABLE 4

Refined anomalous density, occupancy and B-factor for Zn ions in *Pd*AztD and *Ck*AztD.

Protein	Chain	Zn Site	Anomalous σ	Occupancy	B-factor
*Pd*AztD	A	1	7.28	0.71	53.26
		2	3.95	0.81	98.08
	B	1	11.22	0.71	26.28
		2	4.57	0.70	74.44
	C	1	8.70	0.78	41.48
		2	4.78	0.93	97.60
	D	1	9.82	0.72	36.99
		2	15.24	1.00	31.22
*Ck*AztD	A	1	7.01	0.44	36.20
		2	7.58	0.64	37.79
	B	1	12.91	0.69	23.45
		2	5.07	0.92	64.79

Supplementary Table 5. Docked model statistics

Parameter	Pre-Docked Complex (Fig. 8)	*Ab initio* Complex (Fig. S9)
HADDOCK score	-234.2 +/- 8.1	-120.0 +/- 4.6
Cluster size	20	12
RMSD from lowest E structure (Å)	0.3 +/- 0.2	18.8 +/- 0.4
VDW energy (kcal/mol)	-129.6 +/- 5.7	-64.4 +/- 8.3
Electrostatic energy (kcal/mol)	-308.8 +/- 42.6	-124.8 +/- 6.6
Desolvation energy (kcal/mol)	-42.8 +/- 2.8	-34.6 +/- 6.6
Buried surface area (Å^2)	3,359.8 +/- 58.2	1,759.7 +/- 67.5
