# Supplementary material for: Crystal structures of AztD provide mechanistic insights into direct zinc transfer between proteins
Source: Commun Biol. 2019 Aug 9;2:308. doi: 10.1038/s42003-019-0542-z (PMC6689063; doi:10.1038/s42003-019-0542-z)
Supplement: Supplementary file 2 — Description of Additional Supplementary Items [file 42003_2019_542_MOESM2_ESM.docx]

**Description of Additional Supplementary Items**

**Supplementary Data 1.** This is a .sqlite file that can be used to view the genome neighborhood diagrams for all AztD sequences. The file can be uploaded to the Enzyme Function Initiative – Genome Neighborhood Tool under the “View Saved Diagrams” tab at <https://efi.igb.illinois.edu/efi-gnt/>.

**Supplementary Data 2.** The output of the DALI domain database search using *Pd* AztD as a search model.

**Supplementary Data 3.** Source data for all spectroscopic and kinetic figures in .xlsx format.
