# Supplementary material for: Crystal structures of AztD provide mechanistic insights into direct zinc transfer between proteins
Source: Commun Biol. 2019 Aug 9;2:308. doi: 10.1038/s42003-019-0542-z (PMC6689063; doi:10.1038/s42003-019-0542-z)
Supplement: Supplementary file 4 — Supplementary_Data_2 [file 42003_2019_542_MOESM4_ESM.pdf]

Results

Parseable data
Matches to PDB90

- mol1D

Query: mol1D

Select neighbours (check boxes) for viewing as multiple structural alignment or 3D superimposition. The list of neighbours is sorted by Z-score. Similarities with a Z-score lower than 2 are spurious. Each neighbour has links to pairwise structural alignment with the query structure, to pre-computed structural neighbours in the Dali Database, and to the PDB format coordinate file where the neighbour is superimposed onto the query structure.

Structural Alignment | Expand gaps | 3D Superimposition (Jmol Applet) | Sequence Similarity Search | Reset Selection

Summary

No:	Chain	Z	rmsd	lali	nres	%id	PDB	Description
1:	1hj5-B	26.8	3.1	316	559	13	PDB	MOLECULE: NITRITE REDUCTASE;
2:	1gq1-B	26.7	3.1	316	559	13	PDB	MOLECULE: CYTOCHROME CD1 NITRITE REDUCTASE;
3:	1hj4-B	26.7	3.1	316	542	13	PDB	MOLECULE: NITRITE REDUCTASE;
4:	1hzu-A	26.7	3.1	317	521	13	PDB	MOLECULE: NITRITE REDUCTASE;
5:	1qks-A	26.7	3.1	317	559	13	PDB	MOLECULE: CYTOCHROME CD1 NITRITE REDUCTASE;
6:	1dy7-B	26.7	3.1	317	536	13	PDB	MOLECULE: NITRITE REDUCTASE;
7:	1e2r-A	26.6	3.2	316	532	13	PDB	MOLECULE: NITRITE REDUCTASE;
8:	1hj3-B	26.6	3.2	316	542	13	PDB	MOLECULE: NITRITE REDUCTASE;
9:	1h9x-A	26.6	3.1	316	526	13	PDB	MOLECULE: CYTOCHROME CD1 NITRITE REDUCTASE;
10:	1dy7-A	26.6	3.1	316	434	13	PDB	MOLECULE: NITRITE REDUCTASE;
11:	1h9x-B	26.6	3.1	317	529	13	PDB	MOLECULE: CYTOCHROME CD1 NITRITE REDUCTASE;
12:	1aof-A	26.6	3.1	316	532	13	PDB	MOLECULE: NITRITE REDUCTASE;
13:	1e2r-B	26.6	3.1	317	543	12	PDB	MOLECULE: NITRITE REDUCTASE;
14:	1gq1-A	26.5	3.1	316	559	14	PDB	MOLECULE: CYTOCHROME CD1 NITRITE REDUCTASE;
15:	1aoq-B	26.5	3.1	316	559	13	PDB	MOLECULE: NITRITE REDUCTASE;
16:	1hcm-A	26.5	3.1	316	526	13	PDB	MOLECULE: CYTOCHROME CD1 NITRITE REDUCTASE;
17:	1hj5-A	26.5	3.1	316	559	14	PDB	MOLECULE: NITRITE REDUCTASE;
18:	1h9y-A	26.5	3.1	317	520	13	PDB	MOLECULE: CYTOCHROME CD1 NITRITE REDUCTASE;
19:	1n50-A	26.5	3.2	316	538	14	PDB	MOLECULE: NITRITE REDUCTASE;
20:	1n15-A	26.5	3.2	316	538	14	PDB	MOLECULE: NITRITE REDUCTASE;
21:	1aof-B	26.5	3.1	317	542	12	PDB	MOLECULE: NITRITE REDUCTASE;
22:	1hzv-A	26.5	3.1	316	514	13	PDB	MOLECULE: NITRITE REDUCTASE;
23:	1aom-B	26.5	3.2	317	559	13	PDB	MOLECULE: NITRITE REDUCTASE;
24:	1h9y-B	26.4	3.2	315	519	14	PDB	MOLECULE: CYTOCHROME CD1 NITRITE REDUCTASE;
25:	1nno-A	26.4	3.2	316	539	14	PDB	MOLECULE: NITRITE REDUCTASE;
26:	1n50-B	26.4	3.2	316	539	14	PDB	MOLECULE: NITRITE REDUCTASE;
27:	1n90-A	26.4	3.2	316	538	14	PDB	MOLECULE: NITRITE REDUCTASE;
28:	1gjq-B	26.4	3.2	316	539	14	PDB	MOLECULE: NITRITE REDUCTASE;
29:	1nno-B	26.4	3.2	316	539	14	PDB	MOLECULE: NITRITE REDUCTASE;
30:	1n15-B	26.4	3.2	316	539	14	PDB	MOLECULE: NITRITE REDUCTASE;

☐	31:	1n90-B	26.4	3.2	316	539	14 PDB	MOLECULE: NITRITE REDUCTASE;
☐	32:	1hcm-B	26.4	3.1	317	529	13 PDB	MOLECULE: CYTOCHROME CD1 NITRITE REDUCTASE;
☐	33:	1bl9-B	26.4	3.2	316	537	13 PDB	MOLECULE: NITRITE REDUCTASE;
☐	34:	1bl9-A	26.4	3.2	316	537	13 PDB	MOLECULE: NITRITE REDUCTASE;
☐	35:	1hj3-A	26.3	3.1	315	544	13 PDB	MOLECULE: NITRITE REDUCTASE;
☐	36:	1qks-B	26.3	3.2	317	559	13 PDB	MOLECULE: CYTOCHROME CD1 NITRITE REDUCTASE;
☐	37:	1nir-B	26.3	3.2	316	539	14 PDB	MOLECULE: NITRITE REDUCTASE;
☐	38:	1gjq-A	26.3	3.2	316	541	14 PDB	MOLECULE: NITRITE REDUCTASE;
☐	39:	1aom-A	26.2	3.1	316	439	13 PDB	MOLECULE: NITRITE REDUCTASE;
☐	40:	1nir-A	26.2	3.2	316	538	14 PDB	MOLECULE: NITRITE REDUCTASE;
☐	41:	5guw-N	26.2	3.2	316	538	14 PDB	MOLECULE: NITRIC OXIDE REDUCTASE SUBUNIT C;
☐	42:	5guw-M	26.1	3.2	316	538	14 PDB	MOLECULE: NITRIC OXIDE REDUCTASE SUBUNIT C;
☐	43:	4wju-B	25.1	2.9	300	446	8 PDB	MOLECULE: RIBOSOME ASSEMBLY PROTEIN 4;
☐	44:	4wjs-A	25.0	3.0	301	485	8 PDB	MOLECULE: RSA4;
☐	45:	4wju-A	24.6	2.9	297	457	11 PDB	MOLECULE: RIBOSOME ASSEMBLY PROTEIN 4;
☐	46:	4wjv-D	24.5	3.0	297	378	11 PDB	MOLECULE: RIBOSOME ASSEMBLY PROTEIN 4;
☐	47:	4wjv-B	24.5	3.0	297	378	11 PDB	MOLECULE: RIBOSOME ASSEMBLY PROTEIN 4;
☐	48:	4wjv-C	24.5	3.0	297	378	11 PDB	MOLECULE: RIBOSOME ASSEMBLY PROTEIN 4;
☐	49:	4wjv-A	24.4	3.0	297	378	11 PDB	MOLECULE: RIBOSOME ASSEMBLY PROTEIN 4;
☐	50:	4lg9-A	23.4	3.2	292	350	12 PDB	MOLECULE: F-BOX-LIKE/WD REPEAT-CONTAINING PROTE
☐	51:	5nnz-B	23.3	3.2	297	335	8 PDB	MOLECULE: DYNEIN ASSEMBLY FACTOR WITH WDR REPEA
☐	52:	5naf-C	23.3	3.3	291	339	9 PDB	MOLECULE: F-BOX-LIKE/WD REPEAT-CONTAINING PROTE
☐	53:	5naf-A	23.2	3.2	292	352	12 PDB	MOLECULE: F-BOX-LIKE/WD REPEAT-CONTAINING PROTE
☐	54:	5nnz-A	23.1	3.2	297	335	9 PDB	MOLECULE: DYNEIN ASSEMBLY FACTOR WITH WDR REPEA
☐	55:	5naf-B	23.1	3.2	292	347	12 PDB	MOLECULE: F-BOX-LIKE/WD REPEAT-CONTAINING PROTE
☐	56:	5mzh-A	23.1	3.5	298	410	9 PDB	MOLECULE: DYNEIN ASSEMBLY FACTOR WITH WDR REPEA
☐	57:	5mzh-B	23.0	3.3	298	415	8 PDB	MOLECULE: DYNEIN ASSEMBLY FACTOR WITH WDR REPEA
☐	58:	1r5m-A	23.0	3.1	294	351	10 PDB	MOLECULE: SIR4-INTERACTING PROTEIN SIF2;
☐	59:	5naf-D	23.0	3.1	287	331	10 PDB	MOLECULE: F-BOX-LIKE/WD REPEAT-CONTAINING PROTE
☐	60:	4zox-A	22.8	3.5	308	379	10 PDB	MOLECULE: RIBOSOME ASSEMBLY PROTEIN SQT1;
☐	61:	4zov-B	22.8	3.5	308	373	10 PDB	MOLECULE: RIBOSOME ASSEMBLY PROTEIN SQT1;
☐	62:	2ovq-B	22.7	3.1	290	444	11 PDB	MOLECULE: S-PHASE KINASE-ASSOCIATED PROTEIN 1A;
☐	63:	4zov-A	22.7	3.5	308	379	10 PDB	MOLECULE: RIBOSOME ASSEMBLY PROTEIN SQT1;
☐	64:	5v4b-B	22.7	3.1	290	444	11 PDB	MOLECULE: S-PHASE KINASE-ASSOCIATED PROTEIN 1,S
☐	65:	5ams-A	22.6	3.5	308	392	10 PDB	MOLECULE: RIBOSOME ASSEMBLY PROTEIN SQT1;
☐	66:	5ams-B	22.6	3.5	308	379	10 PDB	MOLECULE: RIBOSOME ASSEMBLY PROTEIN SQT1;
☐	67:	1ri6-A	22.6	3.1	280	333	11 PDB	MOLECULE: PUTATIVE ISOMERASE YBHE;
☐	68:	2ovr-B	22.5	3.1	290	442	11 PDB	MOLECULE: S-PHASE KINASE-ASSOCIATED PROTEIN 1A;
☑	69:	1l0q-A	22.5	3.4	270	391	16 PDB	MOLECULE: SURFACE LAYER PROTEIN;
☐	70:	1l0q-D	22.5	3.4	271	391	17 PDB	MOLECULE: SURFACE LAYER PROTEIN;
☐	71:	1l0q-C	22.5	3.4	271	391	17 PDB	MOLECULE: SURFACE LAYER PROTEIN;
☐	72:	1l0q-B	22.4	3.4	271	391	16 PDB	MOLECULE: SURFACE LAYER PROTEIN;
☐	73:	4zoy-A	22.1	4.0	311	406	11 PDB	MOLECULE: SQT1;
☐	74:	4zn4-B	22.0	3.7	308	411	10 PDB	MOLECULE: SQT1;
☐	75:	1nex-D	22.0	3.2	296	444	9 PDB	MOLECULE: CENTROMERE DNA-BINDING PROTEIN COMPLE
☐	76:	3v7d-D	21.9	3.2	297	447	9 PDB	MOLECULE: SUPPRESSOR OF KINETOCHORE PROTEIN 1;
☐	77:	4zn4-A	21.9	3.7	306	409	10 PDB	MOLECULE: SQT1;
☐	78:	1nex-B	21.9	3.2	296	444	9 PDB	MOLECULE: CENTROMERE DNA-BINDING PROTEIN COMPLE
☐	79:	3u4y-B	21.9	3.4	273	317	11 PDB	MOLECULE: UNCHARACTERIZED PROTEIN;
☐	80:	3u4y-A	21.9	3.5	278	319	11 PDB	MOLECULE: UNCHARACTERIZED PROTEIN;
☐	81:	3v7d-B	21.8	3.2	297	450	9 PDB	MOLECULE: SUPPRESSOR OF KINETOCHORE PROTEIN 1;
☐	82:	4zoz-A	21.8	3.6	302	394	11 PDB	MOLECULE: SQT1;
☐	83:	3q7n-A	21.8	3.2	290	355	14 PDB	MOLECULE: LIPOPROTEIN YFGL;
☐	84:	4zoz-B	21.7	3.6	304	394	11 PDB	MOLECULE: SQT1;

85:	2ece-A	21.6	3.2	288	455	10	PDB	MOLECULE: 462AA LONG HYPOTHETICAL SELENIUM-BIND
86:	3iz8-H	21.6	3.0	280	1215	0	PDB	MOLECULE: APAF-1 RELATED KILLER DARK;
87:	1vt4-J	21.6	3.0	280	1215	0	PDB	MOLECULE: APAF-1 RELATED KILLER DARK;
88:	2ovp-B	21.4	3.6	298	441	9	PDB	MOLECULE: S-PHASE KINASE-ASSOCIATED PROTEIN 1A;
89:	3prw-A	21.4	3.2	290	355	14	PDB	MOLECULE: LIPOPROTEIN YFGL;
90:	3p1l-A	21.4	3.3	290	361	14	PDB	MOLECULE: LIPOPROTEIN YFGL;
91:	4qrj-A	21.3	3.5	282	345	10	PDB	MOLECULE: PUTATIVE 6-PHOSPHOGLUCONOLACTONASE;
92:	3mks-B	21.2	3.3	292	444	11	PDB	MOLECULE: SUPPRESSOR OF KINETOCHORE PROTEIN 1;
93:	2yh3-A	21.2	3.3	289	362	14	PDB	MOLECULE: LIPOPROTEIN YFGL;
94:	3scy-A	21.2	3.3	279	356	11	PDB	MOLECULE: HYPOTHETICAL BACTERIAL 6-PHOSPHOGLUCO
95:	3mks-D	21.1	3.4	294	445	11	PDB	MOLECULE: SUPPRESSOR OF KINETOCHORE PROTEIN 1;
96:	3q7o-A	21.1	3.3	289	364	13	PDB	MOLECULE: LIPOPROTEIN YFGL;
97:	3q54-A	21.1	3.3	287	349	14	PDB	MOLECULE: OUTER MEMBRANE ASSEMBLY LIPOPROTEIN Y
98:	1hj4-A	21.1	3.1	317	551	13	PDB	MOLECULE: NITRITE REDUCTASE;
99:	4qrj-B	21.1	3.4	279	343	10	PDB	MOLECULE: PUTATIVE 6-PHOSPHOGLUCONOLACTONASE;
100:	1aoq-A	21.0	3.1	317	551	13	PDB	MOLECULE: NITRITE REDUCTASE;
101:	4xga-A	21.0	3.5	290	347	14	PDB	MOLECULE: OUTER MEMBRANE PROTEIN ASSEMBLY FACTO
102:	5flc-H	21.0	3.1	272	317	11	PDB	MOLECULE: SERINE/THREONINE-PROTEIN KINASE MTOR;
103:	4pk1-A	20.9	3.4	289	494	14	PDB	MOLECULE: CHIMERA PROTEIN OF OUTER MEMBRANE PRO
104:	5ayw-B	20.9	3.5	289	348	13	PDB	MOLECULE: OUTER MEMBRANE PROTEIN ASSEMBLY FACTO
105:	3q7m-A	20.9	3.5	289	344	13	PDB	MOLECULE: LIPOPROTEIN YFGL;
106:	5flc-D	20.9	3.2	272	317	12	PDB	MOLECULE: SERINE/THREONINE-PROTEIN KINASE MTOR;
107:	4jsn-C	20.8	3.1	271	317	11	PDB	MOLECULE: SERINE/THREONINE-PROTEIN KINASE MTOR;
108:	5i5j-A	20.7	3.9	281	531	14	PDB	MOLECULE: NITROUS-OXIDE REDUCTASE;
109:	5i5j-B	20.7	3.9	281	529	14	PDB	MOLECULE: NITROUS-OXIDE REDUCTASE;
110:	3fgb-B	20.6	3.3	274	349	11	PDB	MOLECULE: UNCHARACTERIZED PROTEIN Q89ZH8_BACTN;
111:	3fgb-A	20.6	3.4	277	349	10	PDB	MOLECULE: UNCHARACTERIZED PROTEIN Q89ZH8_BACTN;
112:	5d0o-B	20.5	3.5	290	354	13	PDB	MOLECULE: OUTER MEMBRANE PROTEIN ASSEMBLY FACTO
113:	4hdj-A	20.5	3.4	290	355	16	PDB	MOLECULE: OUTER MEMBRANE PROTEIN ASSEMBLY FACTO
114:	5i5i-A	20.5	3.9	281	539	14	PDB	MOLECULE: NITROUS-OXIDE REDUCTASE;
115:	4o9d-A	20.4	3.4	299	391	9	PDB	MOLECULE: RIK1-ASSOCIATED FACTOR 1;
116:	5i5i-B	20.4	4.0	281	537	14	PDB	MOLECULE: NITROUS-OXIDE REDUCTASE;
117:	4o9d-B	20.3	3.4	297	392	9	PDB	MOLECULE: RIK1-ASSOCIATED FACTOR 1;
118:	4imm-B	20.3	3.5	286	322	9	PDB	MOLECULE: OUTER MEMBRANE ASSEMBLY LIPOPROTEIN Y
119:	4imm-A	20.1	3.4	286	331	10	PDB	MOLECULE: OUTER MEMBRANE ASSEMBLY LIPOPROTEIN Y
120:	1qni-D	20.0	4.0	275	572	13	PDB	MOLECULE: NITROUS-OXIDE REDUCTASE;
121:	1qni-F	20.0	4.1	281	572	14	PDB	MOLECULE: NITROUS-OXIDE REDUCTASE;
122:	1qni-E	19.9	4.0	275	572	13	PDB	MOLECULE: NITROUS-OXIDE REDUCTASE;
123:	1qni-B	19.9	4.2	277	572	13	PDB	MOLECULE: NITROUS-OXIDE REDUCTASE;
124:	3hfq-A	19.9	3.4	273	341	13	PDB	MOLECULE: UNCHARACTERIZED PROTEIN LP_2219;
125:	3sbp-E	19.9	4.1	281	581	14	PDB	MOLECULE: NITROUS-OXIDE REDUCTASE;
126:	1qni-C	19.9	4.2	281	572	14	PDB	MOLECULE: NITROUS-OXIDE REDUCTASE;
127:	3sbr-D	19.8	4.1	281	588	14	PDB	MOLECULE: NITROUS-OXIDE REDUCTASE;
128:	5f75-B	19.7	3.4	288	466	11	PDB	MOLECULE: THIOCYANATE DEHYDROGENASE;
129:	5f30-C	19.7	3.4	288	466	12	PDB	MOLECULE: THIOCYANATE DEHYDROGENASE;
130:	5f30-B	19.7	3.6	291	467	12	PDB	MOLECULE: THIOCYANATE DEHYDROGENASE;
131:	3hfq-B	19.7	3.3	271	341	13	PDB	MOLECULE: UNCHARACTERIZED PROTEIN LP_2219;
132:	5i5m-B	19.7	4.1	280	576	14	PDB	MOLECULE: NITROUS-OXIDE REDUCTASE;
133:	1fwx-B	19.7	4.0	275	589	11	PDB	MOLECULE: NITROUS OXIDE REDUCTASE;
134:	5ljo-B	19.7	3.5	289	371	13	PDB	MOLECULE: OUTER MEMBRANE PROTEIN ASSEMBLY FACTO
135:	3sbq-B	19.7	4.1	281	581	14	PDB	MOLECULE: NITROUS-OXIDE REDUCTASE;
136:	3sbp-F	19.7	4.1	281	581	14	PDB	MOLECULE: NITROUS-OXIDE REDUCTASE;
137:	3sbq-A	19.7	4.1	281	581	14	PDB	MOLECULE: NITROUS-OXIDE REDUCTASE;
138:	3sbp-H	19.7	4.1	281	581	14	PDB	MOLECULE: NITROUS-OXIDE REDUCTASE;

139:	1fwx-D	19.7	4.0	278	588	12	PDB	MOLECULE: NITROUS OXIDE REDUCTASE;
140:	1fwx-A	19.7	4.1	278	591	12	PDB	MOLECULE: NITROUS OXIDE REDUCTASE;
141:	3dsm-A	19.7	3.4	270	327	12	PDB	MOLECULE: UNCHARACTERIZED PROTEIN BACUNI_02894;
142:	5f30-A	19.6	3.4	289	467	12	PDB	MOLECULE: THIOCYANATE DEHYDROGENASE;
143:	5f30-D	19.6	3.4	287	467	12	PDB	MOLECULE: THIOCYANATE DEHYDROGENASE;
144:	5f75-D	19.6	3.4	287	466	12	PDB	MOLECULE: THIOCYANATE DEHYDROGENASE;
145:	2agx-B	19.6	3.4	275	359	12	PDB	MOLECULE: AROMATIC AMINE DEHYDROGENASE;
146:	2h47-D	19.6	3.4	276	361	12	PDB	MOLECULE: AROMATIC AMINE DEHYDROGENASE;
147:	2agy-B	19.6	3.5	274	361	12	PDB	MOLECULE: AROMATIC AMINE DEHYDROGENASE;
148:	2i0r-B	19.6	3.5	275	360	12	PDB	MOLECULE: AROMATIC AMINE DEHYDROGENASE;
149:	2agl-B	19.6	3.4	275	360	12	PDB	MOLECULE: AROMATIC AMINE DEHYDROGENASE;
150:	3sbr-F	19.6	4.1	281	581	14	PDB	MOLECULE: NITROUS-OXIDE REDUCTASE;
151:	5i5m-A	19.6	4.1	280	575	14	PDB	MOLECULE: NITROUS-OXIDE REDUCTASE;
152:	2i0s-B	19.6	3.5	275	360	12	PDB	MOLECULE: AROMATIC AMINE DEHYDROGENASE;
153:	2hjb-A	19.5	3.4	275	358	12	PDB	MOLECULE: AROMATIC AMINE DEHYDROGENASE;
154:	2i0t-B	19.5	3.4	275	361	12	PDB	MOLECULE: AROMATIC AMINE DEHYDROGENASE;
155:	2ah1-B	19.5	3.5	275	360	12	PDB	MOLECULE: AROMATIC AMINE DEHYDROGENASE;
156:	2ok6-B	19.5	3.4	275	360	12	PDB	MOLECULE: AROMATIC AMINE DEHYDROGENASE, SMALL S
157:	2hj4-B	19.5	3.5	274	358	12	PDB	MOLECULE: AROMATIC AMINE DEHYDROGENASE; CHAIN D
158:	2oiz-B	19.5	3.5	275	361	12	PDB	MOLECULE: AROMATIC AMINE DEHYDROGENASE, SMALL S
159:	2agw-B	19.5	3.5	275	361	12	PDB	MOLECULE: AROMATIC AMINE DEHYDROGENASE;
160:	2agy-A	19.5	3.4	275	360	12	PDB	MOLECULE: AROMATIC AMINE DEHYDROGENASE;
161:	2i0s-A	19.5	3.4	275	360	12	PDB	MOLECULE: AROMATIC AMINE DEHYDROGENASE;
162:	2hjb-B	19.5	3.5	274	358	12	PDB	MOLECULE: AROMATIC AMINE DEHYDROGENASE;
163:	2q7q-B	19.5	3.5	274	358	12	PDB	MOLECULE: ARALKYLAMINE DEHYDROGENASE LIGHT CHAI
164:	2iup-B	19.5	3.5	275	361	11	PDB	MOLECULE: AROMATIC AMINE DEHYDROGENASE ALPHA SU
165:	2ah0-B	19.5	3.5	275	361	12	PDB	MOLECULE: AROMATIC AMINE DEHYDROGENASE;
166:	2agw-A	19.5	3.4	275	360	12	PDB	MOLECULE: AROMATIC AMINE DEHYDROGENASE;
167:	2iuv-B	19.5	3.5	274	358	12	PDB	MOLECULE: AROMATIC AMINE DEHYDROGENASE ALPHA SU
168:	2hkr-B	19.5	3.5	275	360	12	PDB	MOLECULE: AROMATIC AMINE DEHYDROGENASE, SMALL S
169:	2hkm-B	19.5	3.5	275	360	12	PDB	MOLECULE: AROMATIC AMINE DEHYDROGENASE;
170:	2hxc-B	19.5	3.5	275	360	12	PDB	MOLECULE: AROMATIC AMINE DEHYDROGENASE;
171:	2i0r-A	19.5	3.4	275	360	12	PDB	MOLECULE: AROMATIC AMINE DEHYDROGENASE;
172:	2h47-F	19.4	3.6	274	362	12	PDB	MOLECULE: AROMATIC AMINE DEHYDROGENASE;
173:	2iuq-B	19.4	3.5	276	361	12	PDB	MOLECULE: AROMATIC AMINE DEHYDROGENASE ALPHA SU
174:	2agl-A	19.4	3.5	275	360	12	PDB	MOLECULE: AROMATIC AMINE DEHYDROGENASE;
175:	2ok4-B	19.4	3.5	276	361	12	PDB	MOLECULE: AROMATIC AMINE DEHYDROGENASE, SMALL S
176:	2i0t-A	19.4	3.4	275	359	12	PDB	MOLECULE: AROMATIC AMINE DEHYDROGENASE;
177:	2hxc-A	19.4	3.4	275	359	12	PDB	MOLECULE: AROMATIC AMINE DEHYDROGENASE;
178:	2oiz-A	19.4	3.4	275	360	12	PDB	MOLECULE: AROMATIC AMINE DEHYDROGENASE, SMALL S
179:	2h3x-A	19.4	3.5	275	359	12	PDB	MOLECULE: AROMATIC AMINE DEHYDROGENASE;
180:	2ojy-B	19.4	3.5	274	361	12	PDB	MOLECULE: AROMATIC AMINE DEHYDROGENASE, SMALL S
181:	2iur-B	19.4	3.5	273	361	11	PDB	MOLECULE: AROMATIC AMINE DEHYDROGENASE ALPHA SU
182:	2h47-H	19.4	3.5	274	362	12	PDB	MOLECULE: AROMATIC AMINE DEHYDROGENASE;
183:	2iup-A	19.4	3.5	274	360	12	PDB	MOLECULE: AROMATIC AMINE DEHYDROGENASE ALPHA SU
184:	2iuv-A	19.4	3.5	275	359	12	PDB	MOLECULE: AROMATIC AMINE DEHYDROGENASE ALPHA SU
185:	2hkr-A	19.4	3.5	274	360	12	PDB	MOLECULE: AROMATIC AMINE DEHYDROGENASE, SMALL S
186:	2iuq-A	19.4	3.5	275	360	12	PDB	MOLECULE: AROMATIC AMINE DEHYDROGENASE ALPHA SU
187:	2ah1-A	19.4	3.5	273	359	11	PDB	MOLECULE: AROMATIC AMINE DEHYDROGENASE;
188:	5f75-C	19.3	3.4	288	475	12	PDB	MOLECULE: THIOCYANATE DEHYDROGENASE;
189:	5f75-A	19.3	3.4	288	474	12	PDB	MOLECULE: THIOCYANATE DEHYDROGENASE;
190:	2hkm-A	19.3	3.4	275	360	12	PDB	MOLECULE: AROMATIC AMINE DEHYDROGENASE;
191:	2ok6-A	19.3	3.5	275	359	12	PDB	MOLECULE: AROMATIC AMINE DEHYDROGENASE, SMALL S
192:	2ok4-A	19.3	3.5	275	360	12	PDB	MOLECULE: AROMATIC AMINE DEHYDROGENASE, SMALL S

193:	2ojy-A	19.3	3.5	275	360	12	PDB	MOLECULE: AROMATIC AMINE DEHYDROGENASE, SMALL S
194:	2hj4-A	19.3	3.5	273	357	12	PDB	MOLECULE: AROMATIC AMINE DEHYDROGENASE; CHAIN D
195:	2agz-B	19.3	3.5	275	361	12	PDB	MOLECULE: AROMATIC AMINE DEHYDROGENASE;
196:	2agx-A	19.3	3.4	275	359	12	PDB	MOLECULE: AROMATIC AMINE DEHYDROGENASE;
197:	2ah0-A	19.3	3.5	275	360	12	PDB	MOLECULE: AROMATIC AMINE DEHYDROGENASE;
198:	2agz-A	19.3	3.5	274	361	12	PDB	MOLECULE: AROMATIC AMINE DEHYDROGENASE;
199:	2iaa-D	19.3	3.4	274	359	12	PDB	MOLECULE: AROMATIC AMINE DEHYDROGENASE;
200:	2h47-A	19.3	3.5	272	360	12	PDB	MOLECULE: AROMATIC AMINE DEHYDROGENASE;
201:	2iur-A	19.3	3.5	273	360	11	PDB	MOLECULE: AROMATIC AMINE DEHYDROGENASE ALPHA SU
202:	2q7q-A	19.3	3.4	274	356	12	PDB	MOLECULE: ARALKYLAMINE DEHYDROGENASE LIGHT CHAI
203:	2iwk-B	19.3	4.0	276	590	12	PDB	MOLECULE: NITROUS OXIDE REDUCTASE;
204:	2h3x-D	19.2	3.5	272	359	12	PDB	MOLECULE: AROMATIC AMINE DEHYDROGENASE;
205:	1qni-A	19.2	4.2	281	572	14	PDB	MOLECULE: NITROUS-OXIDE REDUCTASE;
206:	2iwk-A	19.2	4.0	276	590	12	PDB	MOLECULE: NITROUS OXIDE REDUCTASE;
207:	2iwf-B	19.2	4.1	276	590	12	PDB	MOLECULE: NITROUS-OXIDE REDUCTASE;
208:	2iwf-A	19.2	4.1	276	590	12	PDB	MOLECULE: NITROUS-OXIDE REDUCTASE;
209:	2iaa-A	19.1	3.5	273	359	11	PDB	MOLECULE: AROMATIC AMINE DEHYDROGENASE;
210:	3sbp-C	19.0	4.1	281	581	14	PDB	MOLECULE: NITROUS-OXIDE REDUCTASE;
211:	3sbp-A	18.8	4.1	281	581	14	PDB	MOLECULE: NITROUS-OXIDE REDUCTASE;
212:	3sbp-B	18.8	4.1	281	581	14	PDB	MOLECULE: NITROUS-OXIDE REDUCTASE;
213:	3sbr-E	18.8	4.1	281	581	14	PDB	MOLECULE: NITROUS-OXIDE REDUCTASE;
214:	3sbr-H	18.8	4.1	281	581	14	PDB	MOLECULE: NITROUS-OXIDE REDUCTASE;
215:	3sbr-B	18.8	4.1	281	581	14	PDB	MOLECULE: NITROUS-OXIDE REDUCTASE;
216:	3sbr-C	18.8	4.1	281	581	14	PDB	MOLECULE: NITROUS-OXIDE REDUCTASE;
217:	3sbp-G	18.7	4.1	281	585	14	PDB	MOLECULE: NITROUS-OXIDE REDUCTASE;
218:	3sbr-A	18.7	4.1	281	581	14	PDB	MOLECULE: NITROUS-OXIDE REDUCTASE;
219:	5h64-C	18.7	3.2	265	317	10	PDB	MOLECULE: SERINE/THREONINE-PROTEIN KINASE MTOR;
220:	5h64-c	18.7	3.3	266	317	10	PDB	MOLECULE: SERINE/THREONINE-PROTEIN KINASE MTOR;
221:	2gc4-A	18.6	3.7	274	382	14	PDB	MOLECULE: METHYLAMINE DEHYDROGENASE HEAVY CHAIN
222:	3sbp-D	18.6	4.1	281	588	14	PDB	MOLECULE: NITROUS-OXIDE REDUCTASE;
223:	3rmz-F	18.6	3.8	273	376	13	PDB	MOLECULE: METHYLAMINE UTILIZATION PROTEIN MAUG;
224:	3sbr-G	18.6	4.1	281	585	14	PDB	MOLECULE: NITROUS-OXIDE REDUCTASE;
225:	2bbk-H	18.6	3.8	275	355	12	PDB	MOLECULE: METHYLAMINE DEHYDROGENASE (HEAVY SUBU
226:	2gc7-I	18.6	3.7	276	382	13	PDB	MOLECULE: METHYLAMINE DEHYDROGENASE HEAVY CHAIN
227:	3orv-F	18.6	3.8	274	376	13	PDB	MOLECULE: METHYLAMINE UTILIZATION PROTEIN MAUG;
228:	1jju-B	18.6	3.6	267	337	9	PDB	MOLECULE: QUINOHEMOPROTEIN AMINE DEHYDROGENASE;
229:	1pby-B	18.6	3.6	266	337	9	PDB	MOLECULE: QUINOHEMOPROTEIN AMINE DEHYDROGENASE
230:	3vgz-A	18.6	3.2	254	321	15	PDB	MOLECULE: UNCHARACTERIZED PROTEIN YNCE;
231:	3l4m-F	18.5	3.7	273	376	13	PDB	MOLECULE: METHYLAMINE UTILIZATION PROTEIN MAUG;
232:	3rn1-F	18.5	3.8	278	376	12	PDB	MOLECULE: METHYLAMINE UTILIZATION PROTEIN MAUG;
233:	4fav-D	18.5	3.8	274	376	12	PDB	MOLECULE: METHYLAMINE UTILIZATION PROTEIN MAUG;
234:	2gc4-E	18.5	3.7	276	382	13	PDB	MOLECULE: METHYLAMINE DEHYDROGENASE HEAVY CHAIN
235:	3orv-D	18.5	3.8	275	376	12	PDB	MOLECULE: METHYLAMINE UTILIZATION PROTEIN MAUG;
236:	3pxw-F	18.5	3.8	276	376	12	PDB	MOLECULE: METHYLAMINE UTILIZATION PROTEIN MAUG;
237:	2gc7-E	18.5	3.7	277	382	13	PDB	MOLECULE: METHYLAMINE DEHYDROGENASE HEAVY CHAIN
238:	2j57-I	18.5	3.8	275	382	12	PDB	MOLECULE: METHYLAMINE DEHYDROGENASE HEAVY CHAIN
239:	3sle-D	18.5	3.7	272	376	14	PDB	MOLECULE: METHYLAMINE UTILIZATION PROTEIN MAUG;
240:	1mg2-M	18.5	3.7	274	382	13	PDB	MOLECULE: METHYLAMINE DEHYDROGENASE, HEAVY CHAI
241:	2gc4-I	18.5	3.7	277	382	12	PDB	MOLECULE: METHYLAMINE DEHYDROGENASE HEAVY CHAIN
242:	3svw-F	18.5	3.8	277	376	12	PDB	MOLECULE: METHYLAMINE UTILIZATION PROTEIN MAUG;
243:	2gc7-M	18.5	3.7	277	382	13	PDB	MOLECULE: METHYLAMINE DEHYDROGENASE HEAVY CHAIN
244:	1mg3-I	18.5	3.7	277	382	13	PDB	MOLECULE: METHYLAMINE DEHYDROGENASE, HEAVY CHAI
245:	2gc4-M	18.5	3.7	277	382	13	PDB	MOLECULE: METHYLAMINE DEHYDROGENASE HEAVY CHAIN
246:	3pxs-F	18.5	3.8	274	376	13	PDB	MOLECULE: METHYLAMINE UTILIZATION PROTEIN MAUG;

☐	247:	3rmz-D	18.5	3.9	274	376	13 PDB	MOLECULE: METHYLAMINE UTILIZATION PROTEIN MAUG;
☐	248:	3sxt-F	18.5	3.9	274	376	13 PDB	MOLECULE: METHYLAMINE UTILIZATION PROTEIN MAUG;
☐	249:	2j56-H	18.5	3.8	278	375	12 PDB	MOLECULE: METHYLAMINE DEHYDROGENASE HEAVY CHAIN
☐	250:	1mg3-E	18.5	3.7	275	382	13 PDB	MOLECULE: METHYLAMINE DEHYDROGENASE, HEAVY CHAI
☐	251:	3l4o-F	18.5	3.8	275	376	12 PDB	MOLECULE: METHYLAMINE UTILIZATION PROTEIN MAUG;
☐	252:	4fb1-D	18.5	3.7	275	376	12 PDB	MOLECULE: METHYLAMINE UTILIZATION PROTEIN MAUG;
☐	253:	3svw-D	18.5	3.9	274	376	13 PDB	MOLECULE: METHYLAMINE UTILIZATION PROTEIN MAUG;
☐	254:	4y5r-F	18.4	3.8	276	376	12 PDB	MOLECULE: METHYLAMINE UTILIZATION PROTEIN MAUG;
☐	255:	2j57-H	18.4	3.8	278	382	12 PDB	MOLECULE: METHYLAMINE DEHYDROGENASE HEAVY CHAIN
☐	256:	4k3i-F	18.4	3.8	274	376	14 PDB	MOLECULE: METHYLAMINE UTILIZATION PROTEIN MAUG;
☐	257:	4fav-F	18.4	3.8	277	377	12 PDB	MOLECULE: METHYLAMINE UTILIZATION PROTEIN MAUG;
☐	258:	2j56-J	18.4	3.8	278	375	12 PDB	MOLECULE: METHYLAMINE DEHYDROGENASE HEAVY CHAIN
☐	259:	3rlm-F	18.4	3.8	277	376	13 PDB	MOLECULE: METHYLAMINE UTILIZATION PROTEIN MAUG;
☐	260:	1fwx-C	18.4	4.0	275	590	11 PDB	MOLECULE: NITROUS OXIDE REDUCTASE;
☐	261:	2bbk-J	18.4	3.7	277	355	12 PDB	MOLECULE: METHYLAMINE DEHYDROGENASE (HEAVY SUBU
☐	262:	3sjl-D	18.4	3.7	274	376	13 PDB	MOLECULE: METHYLAMINE UTILIZATION PROTEIN MAUG;
☐	263:	1mg2-I	18.4	3.7	276	382	13 PDB	MOLECULE: METHYLAMINE DEHYDROGENASE, HEAVY CHAI
☐	264:	4fa5-F	18.4	3.8	273	376	13 PDB	MOLECULE: METHYLAMINE UTILIZATION PROTEIN MAUG;
☐	265:	3sle-F	18.4	3.8	274	376	13 PDB	MOLECULE: METHYLAMINE UTILIZATION PROTEIN MAUG;
☐	266:	3pxt-F	18.4	3.8	276	376	12 PDB	MOLECULE: METHYLAMINE UTILIZATION PROTEIN MAUG;
☐	267:	4y5r-D	18.4	3.8	275	376	12 PDB	MOLECULE: METHYLAMINE UTILIZATION PROTEIN MAUG;
☐	268:	2mta-H	18.4	3.8	276	373	13 PDB	MOLECULE: METHYLAMINE DEHYDROGENASE (HEAVY SUBU
☐	269:	2gc7-A	18.4	3.7	275	382	12 PDB	MOLECULE: METHYLAMINE DEHYDROGENASE HEAVY CHAIN
☐	270:	3sws-F	18.4	3.8	275	376	12 PDB	MOLECULE: METHYLAMINE UTILIZATION PROTEIN MAUG;
☐	271:	2j55-J	18.4	3.6	275	381	13 PDB	MOLECULE: METHYLAMINE DEHYDROGENASE HEAVY CHAIN
☐	272:	3sjl-F	18.4	3.8	276	376	12 PDB	MOLECULE: METHYLAMINE UTILIZATION PROTEIN MAUG;
☐	273:	4fa1-F	18.4	3.8	276	376	12 PDB	MOLECULE: METHYLAMINE UTILIZATION PROTEIN MAUG;
☐	274:	2j57-J	18.4	3.8	278	382	12 PDB	MOLECULE: METHYLAMINE DEHYDROGENASE HEAVY CHAIN
☐	275:	4fan-F	18.4	3.8	275	376	12 PDB	MOLECULE: METHYLAMINE UTILIZATION PROTEIN MAUG;
☐	276:	2j55-H	18.4	3.8	277	382	12 PDB	MOLECULE: METHYLAMINE DEHYDROGENASE HEAVY CHAIN
☐	277:	3l4m-D	18.4	3.7	274	376	13 PDB	MOLECULE: METHYLAMINE UTILIZATION PROTEIN MAUG;
☐	278:	3pxw-D	18.4	3.8	278	376	12 PDB	MOLECULE: METHYLAMINE UTILIZATION PROTEIN MAUG;
☐	279:	1mg2-A	18.4	3.8	278	382	12 PDB	MOLECULE: METHYLAMINE DEHYDROGENASE, HEAVY CHAI
☐	280:	3l4o-D	18.4	3.7	275	376	13 PDB	MOLECULE: METHYLAMINE UTILIZATION PROTEIN MAUG;
☐	281:	3rlm-D	18.4	3.8	275	376	12 PDB	MOLECULE: METHYLAMINE UTILIZATION PROTEIN MAUG;
☐	282:	4k3i-D	18.4	3.8	273	376	13 PDB	MOLECULE: METHYLAMINE UTILIZATION PROTEIN MAUG;
☐	283:	4fa9-F	18.4	3.8	273	376	13 PDB	MOLECULE: METHYLAMINE UTILIZATION PROTEIN MAUG;
☐	284:	3sws-D	18.4	3.8	275	376	13 PDB	MOLECULE: METHYLAMINE UTILIZATION PROTEIN MAUG;
☐	285:	1mg3-A	18.4	3.7	275	382	13 PDB	MOLECULE: METHYLAMINE DEHYDROGENASE, HEAVY CHAI
☐	286:	1mg2-E	18.4	3.8	278	382	12 PDB	MOLECULE: METHYLAMINE DEHYDROGENASE, HEAVY CHAI
☐	287:	4fa9-D	18.4	3.8	278	376	12 PDB	MOLECULE: METHYLAMINE UTILIZATION PROTEIN MAUG;
☐	288:	5i2t-A	18.3	3.2	251	606	11 PDB	MOLECULE: PERIODIC TRYPTOPHAN PROTEIN 2;
☐	289:	1mg3-M	18.3	3.7	275	382	12 PDB	MOLECULE: METHYLAMINE DEHYDROGENASE, HEAVY CHAI
☐	290:	3rn0-D	18.3	3.8	276	376	12 PDB	MOLECULE: METHYLAMINE UTILIZATION PROTEIN MAUG;
☐	291:	2j57-G	18.3	3.8	276	382	12 PDB	MOLECULE: METHYLAMINE DEHYDROGENASE HEAVY CHAIN
☐	292:	4fa4-D	18.3	3.8	277	376	11 PDB	MOLECULE: METHYLAMINE UTILIZATION PROTEIN MAUG;
☐	293:	4fa5-D	18.3	3.8	278	376	12 PDB	MOLECULE: METHYLAMINE UTILIZATION PROTEIN MAUG;
☐	294:	4o1q-F	18.3	3.8	273	376	13 PDB	MOLECULE: METHYLAMINE UTILIZATION PROTEIN MAUG;
☐	295:	4fan-D	18.3	3.8	276	376	12 PDB	MOLECULE: METHYLAMINE UTILIZATION PROTEIN MAUG;
☐	296:	3rn1-D	18.3	3.8	276	376	12 PDB	MOLECULE: METHYLAMINE UTILIZATION PROTEIN MAUG;
☐	297:	4fa4-F	18.3	3.9	277	376	11 PDB	MOLECULE: METHYLAMINE UTILIZATION PROTEIN MAUG;
☐	298:	3rn0-F	18.3	3.8	275	376	12 PDB	MOLECULE: METHYLAMINE UTILIZATION PROTEIN MAUG;
☐	299:	3pxt-D	18.3	3.8	278	376	12 PDB	MOLECULE: METHYLAMINE UTILIZATION PROTEIN MAUG;
☐	300:	3sxt-D	18.3	3.9	277	375	12 PDB	MOLECULE: METHYLAMINE UTILIZATION PROTEIN MAUG;

301:	4fa1-D	18.3	3.8	278	376	12	PDB	MOLECULE: METHYLAMINE UTILIZATION PROTEIN MAUG;
302:	4fb1-F	18.3	3.9	277	376	12	PDB	MOLECULE: METHYLAMINE UTILIZATION PROTEIN MAUG;
303:	3vgz-D	18.3	3.2	252	321	15	PDB	MOLECULE: UNCHARACTERIZED PROTEIN YNCE;
304:	3c75-H	18.2	3.8	278	375	10	PDB	MOLECULE: METHYLAMINE DEHYDROGENASE HEAVY CHAIN
305:	3pxs-D	18.2	3.8	277	376	12	PDB	MOLECULE: METHYLAMINE UTILIZATION PROTEIN MAUG;
306:	4o1q-D	18.2	3.8	277	376	13	PDB	MOLECULE: METHYLAMINE UTILIZATION PROTEIN MAUG;
307:	3c75-J	18.2	3.8	278	375	10	PDB	MOLECULE: METHYLAMINE DEHYDROGENASE HEAVY CHAIN
308:	1mda-H	18.2	3.6	270	368	10	PDB	MOLECULE: METHYLAMINE DEHYDROGENASE (HEAVY SUBU
309:	1jmx-B	18.2	3.9	271	339	10	PDB	MOLECULE: AMINE DEHYDROGENASE;
310:	1jmz-B	18.2	3.9	271	339	10	PDB	MOLECULE: AMINE DEHYDROGENASE;
311:	3vh0-A	18.2	3.3	253	322	15	PDB	MOLECULE: UNCHARACTERIZED PROTEIN YNCE;
312:	5sum-A	18.1	4.3	285	391	11	PDB	MOLECULE: RIBOSOME BIOGENESIS PROTEIN NSA1;
313:	5sum-B	18.1	4.3	285	391	11	PDB	MOLECULE: RIBOSOME BIOGENESIS PROTEIN NSA1;
314:	5jpq-M	18.0	3.6	268	309	0	PDB	MOLECULE: WD40 DOMAIN PROTEINS;
315:	5jpq-O	18.0	3.6	268	309	0	PDB	MOLECULE: WD40 DOMAIN PROTEINS;
316:	5c2v-E	18.0	3.3	256	352	13	PDB	MOLECULE: HYDRAZINE SYNTHASE ALPHA SUBUNIT;
317:	1mda-J	18.0	3.6	268	368	10	PDB	MOLECULE: METHYLAMINE DEHYDROGENASE (HEAVY SUBU
318:	2z2n-A	17.9	3.6	257	293	9	PDB	MOLECULE: VIRGINIAMYCIN B LYASE;
319:	3ow8-C	17.8	3.1	253	300	11	PDB	MOLECULE: WD REPEAT-CONTAINING PROTEIN 61;
320:	3ow8-D	17.8	3.1	253	300	11	PDB	MOLECULE: WD REPEAT-CONTAINING PROTEIN 61;
321:	4nsx-A	17.8	3.7	273	634	11	PDB	MOLECULE: U3 SMALL NUCLEOLAR RNA-ASSOCIATED PRO
322:	5c2w-B	17.8	3.3	255	352	13	PDB	MOLECULE: HYDRAZINE SYNTHASE ALPHA SUBUNIT;
323:	4a0l-D	17.8	3.3	263	355	8	PDB	MOLECULE: DNA DAMAGE-BINDING PROTEIN 1;
324:	4a0l-B	17.8	3.3	263	355	8	PDB	MOLECULE: DNA DAMAGE-BINDING PROTEIN 1;
325:	5h3t-A	17.7	3.3	262	652	11	PDB	MOLECULE: GEM-ASSOCIATED PROTEIN 5;
326:	5flc-E	17.7	3.2	263	1029	0	PDB	MOLECULE: SERINE/THREONINE-PROTEIN KINASE MTOR;
327:	3bws-A	17.7	3.5	252	407	12	PDB	MOLECULE: PROTEIN LP49;
328:	5h3t-D	17.7	3.3	262	657	11	PDB	MOLECULE: GEM-ASSOCIATED PROTEIN 5;
329:	4r5o-C	17.7	3.6	262	416	11	PDB	MOLECULE: QUINONPROTEIN ALCOHOL DEHYDROGENASE-L
330:	5h1j-A	17.6	3.4	259	693	11	PDB	MOLECULE: GEM-ASSOCIATED PROTEIN 5;
331:	5h1k-A	17.6	3.6	262	674	12	PDB	MOLECULE: GEM-ASSOCIATED PROTEIN 5;
332:	4r5o-A	17.6	3.6	261	426	11	PDB	MOLECULE: QUINONPROTEIN ALCOHOL DEHYDROGENASE-L
333:	5tee-A	17.6	3.3	261	686	11	PDB	MOLECULE: GEM-ASSOCIATED PROTEIN 5;
334:	5tef-A	17.6	3.4	262	678	11	PDB	MOLECULE: GEM-ASSOCIATED PROTEIN 5;
335:	4r5o-B	17.6	3.6	262	416	11	PDB	MOLECULE: QUINONPROTEIN ALCOHOL DEHYDROGENASE-L
336:	5h3t-B	17.6	3.3	260	652	11	PDB	MOLECULE: GEM-ASSOCIATED PROTEIN 5;
337:	5c2w-E	17.6	3.3	256	352	13	PDB	MOLECULE: HYDRAZINE SYNTHASE ALPHA SUBUNIT;
338:	3ow8-A	17.6	3.2	252	293	11	PDB	MOLECULE: WD REPEAT-CONTAINING PROTEIN 61;
339:	4bh6-C	17.6	3.7	261	304	12	PDB	MOLECULE: APC/C ACTIVATOR PROTEIN CDH1;
340:	4bh6-E	17.6	3.7	261	303	12	PDB	MOLECULE: APC/C ACTIVATOR PROTEIN CDH1;
341:	4bh6-H	17.6	3.8	261	299	12	PDB	MOLECULE: APC/C ACTIVATOR PROTEIN CDH1;
342:	5a31-R	17.5	3.7	260	386	12	PDB	MOLECULE: ANAPHASE-PROMOTING COMPLEX SUBUNIT 1;
343:	5h3s-A	17.5	3.3	263	655	11	PDB	MOLECULE: GEM-ASSOCIATED PROTEIN 5;
344:	5h3t-C	17.5	3.3	261	657	11	PDB	MOLECULE: GEM-ASSOCIATED PROTEIN 5;
345:	5gxh-A	17.5	3.3	262	672	11	PDB	MOLECULE: GEM-ASSOCIATED PROTEIN 5;
346:	5h1l-A	17.5	3.5	262	682	12	PDB	MOLECULE: GEM-ASSOCIATED PROTEIN 5;
347:	5h1m-A	17.5	3.5	262	674	12	PDB	MOLECULE: GEM-ASSOCIATED PROTEIN 5;
348:	4r5o-D	17.5	3.6	262	425	11	PDB	MOLECULE: QUINONPROTEIN ALCOHOL DEHYDROGENASE-L
349:	3bws-B	17.5	3.6	252	407	12	PDB	MOLECULE: PROTEIN LP49;
350:	4bh6-B	17.5	3.7	261	304	12	PDB	MOLECULE: APC/C ACTIVATOR PROTEIN CDH1;
351:	4bh6-D	17.5	3.7	261	305	12	PDB	MOLECULE: APC/C ACTIVATOR PROTEIN CDH1;
352:	4bh6-F	17.5	3.7	261	304	12	PDB	MOLECULE: APC/C ACTIVATOR PROTEIN CDH1;
353:	4bh6-G	17.5	3.7	261	303	12	PDB	MOLECULE: APC/C ACTIVATOR PROTEIN CDH1;
354:	4bh6-A	17.5	3.7	261	304	12	PDB	MOLECULE: APC/C ACTIVATOR PROTEIN CDH1;

355:	5tha-A	17.4	3.4	263	681	11	PDB	MOLECULE: GEM-ASSOCIATED PROTEIN 5;
356:	5h3u-B	17.4	3.3	262	662	11	PDB	MOLECULE: GEM-ASSOCIATED PROTEIN 5;
357:	5h3s-B	17.4	3.3	261	645	11	PDB	MOLECULE: GEM-ASSOCIATED PROTEIN 5;
358:	3b7f-A	17.4	3.6	267	368	10	PDB	MOLECULE: GLYCOSYL HYDROLASE, BNR REPEAT;
359:	1vyh-G	17.4	3.5	257	310	6	PDB	MOLECULE: PLATELET-ACTIVATING FACTOR ACETYLHYDR
360:	2z2o-D	17.4	3.7	257	295	9	PDB	MOLECULE: VIRGINIAMYCIN B LYASE;
361:	2z2p-A	17.4	3.6	255	293	9	PDB	MOLECULE: VIRGINIAMYCIN B LYASE;
362:	2z2o-B	17.4	3.7	255	293	9	PDB	MOLECULE: VIRGINIAMYCIN B LYASE;
363:	2z2p-B	17.4	3.6	255	293	9	PDB	MOLECULE: VIRGINIAMYCIN B LYASE;
364:	5wve-I	17.4	4.0	282	1144	10	PDB	MOLECULE: APOPTOTIC PROTEASE-ACTIVATING FACTOR
365:	5wve-G	17.4	4.0	282	1144	10	PDB	MOLECULE: APOPTOTIC PROTEASE-ACTIVATING FACTOR
366:	5wve-C	17.4	4.0	278	1144	10	PDB	MOLECULE: APOPTOTIC PROTEASE-ACTIVATING FACTOR
367:	5wve-K	17.4	4.0	278	1144	9	PDB	MOLECULE: APOPTOTIC PROTEASE-ACTIVATING FACTOR
368:	1vyh-P	17.4	3.5	257	310	6	PDB	MOLECULE: PLATELET-ACTIVATING FACTOR ACETYLHYDR
369:	4cy3-A	17.4	3.4	251	303	11	PDB	MOLECULE: PROTEIN WILL DIE SLOWLY;
370:	5m23-A	17.4	3.4	252	301	11	PDB	MOLECULE: WD REPEAT-CONTAINING PROTEIN 5;
371:	3ow8-B	17.3	3.2	251	292	11	PDB	MOLECULE: WD REPEAT-CONTAINING PROTEIN 61;
372:	5gxi-A	17.3	3.4	262	667	11	PDB	MOLECULE: GEM-ASSOCIATED PROTEIN 5;
373:	5wve-A	17.3	4.0	281	1144	10	PDB	MOLECULE: APOPTOTIC PROTEASE-ACTIVATING FACTOR
374:	5wve-E	17.3	4.0	279	1144	10	PDB	MOLECULE: APOPTOTIC PROTEASE-ACTIVATING FACTOR
375:	5wve-M	17.3	4.0	281	1144	10	PDB	MOLECULE: APOPTOTIC PROTEASE-ACTIVATING FACTOR
376:	1vyh-L	17.3	3.5	257	310	6	PDB	MOLECULE: PLATELET-ACTIVATING FACTOR ACETYLHYDR
377:	1vyh-D	17.3	3.5	256	310	6	PDB	MOLECULE: PLATELET-ACTIVATING FACTOR ACETYLHYDR
378:	1vyh-K	17.3	3.5	256	310	6	PDB	MOLECULE: PLATELET-ACTIVATING FACTOR ACETYLHYDR
379:	4qqe-A	17.3	3.5	253	304	10	PDB	MOLECULE: WD REPEAT-CONTAINING PROTEIN 5;
380:	4cy1-B	17.3	3.4	252	301	10	PDB	MOLECULE: WD REPEAT-CONTAINING PROTEIN 5;
381:	4cy2-A	17.3	3.5	253	304	11	PDB	MOLECULE: WD REPEAT-CONTAINING PROTEIN 5;
382:	4cy1-A	17.3	3.4	253	303	10	PDB	MOLECULE: WD REPEAT-CONTAINING PROTEIN 5;
383:	4o45-A	17.3	3.4	253	305	10	PDB	MOLECULE: WD REPEAT-CONTAINING PROTEIN 5;
384:	3mmy-E	17.2	4.0	273	354	12	PDB	MOLECULE: MRNA EXPORT FACTOR;
385:	2z2o-A	17.2	3.7	255	298	8	PDB	MOLECULE: VIRGINIAMYCIN B LYASE;
386:	3mmy-A	17.2	4.0	273	354	12	PDB	MOLECULE: MRNA EXPORT FACTOR;
387:	1vyh-C	17.2	3.5	257	310	6	PDB	MOLECULE: PLATELET-ACTIVATING FACTOR ACETYLHYDR
388:	5fvm-D	17.2	3.2	264	299	10	PDB	MOLECULE: SERINE/THREONINE-PROTEIN KINASE TOR2;
389:	1vyh-O	17.2	3.5	257	310	6	PDB	MOLECULE: PLATELET-ACTIVATING FACTOR ACETYLHYDR
390:	5eal-A	17.2	3.4	252	304	11	PDB	MOLECULE: WD REPEAT-CONTAINING PROTEIN 5;
391:	4y7r-A	17.2	3.5	254	304	10	PDB	MOLECULE: WD REPEAT-CONTAINING PROTEIN 5;
392:	2co0-C	17.2	3.4	253	304	10	PDB	MOLECULE: WD-REPEAT PROTEIN 5;
393:	5eal-B	17.2	3.5	254	304	10	PDB	MOLECULE: WD REPEAT-CONTAINING PROTEIN 5;
394:	4cy5-A	17.2	3.5	253	303	11	PDB	MOLECULE: PROTEIN WILL DIE SLOWLY;
395:	1vyh-H	17.2	3.5	256	310	6	PDB	MOLECULE: PLATELET-ACTIVATING FACTOR ACETYLHYDR
396:	4ia9-A	17.2	3.5	252	305	11	PDB	MOLECULE: WD REPEAT-CONTAINING PROTEIN 5;
397:	3zwl-B	17.1	3.4	256	339	11	PDB	MOLECULE: EUKARYOTIC TRANSLATION INITIATION FAC
398:	2z2o-C	17.1	3.8	256	299	9	PDB	MOLECULE: VIRGINIAMYCIN B LYASE;
399:	2qc5-A	17.1	3.7	255	298	9	PDB	MOLECULE: STREPTOGRAMIN B LACTONASE;
400:	3vh0-C	17.1	4.2	269	321	13	PDB	MOLECULE: UNCHARACTERIZED PROTEIN YNCE;
401:	3vh0-B	17.1	4.2	269	321	13	PDB	MOLECULE: UNCHARACTERIZED PROTEIN YNCE;
402:	4owr-A	17.1	3.9	265	331	12	PDB	MOLECULE: MRNA EXPORT FACTOR;
403:	4yd8-B	17.1	3.0	240	334	8	PDB	MOLECULE: PROTEIN PTHB1;
404:	5eam-B	17.1	3.4	253	304	10	PDB	MOLECULE: WD REPEAT-CONTAINING PROTEIN 5;
405:	2cnx-A	17.1	3.5	253	306	10	PDB	MOLECULE: WD-REPEAT PROTEIN 5;
406:	4gmb-A	17.1	3.5	254	307	11	PDB	MOLECULE: WD REPEAT-CONTAINING PROTEIN 5;
407:	5ear-B	17.1	3.5	252	301	11	PDB	MOLECULE: WD REPEAT-CONTAINING PROTEIN 5;
408:	3mmy-C	17.0	4.0	273	355	12	PDB	MOLECULE: MRNA EXPORT FACTOR;

409:	3vgz-C	17.0	4.2	268	323	13	PDB	MOLECULE: UNCHARACTERIZED PROTEIN YNCE;
410:	3j80-g	17.0	3.7	257	318	10	PDB	MOLECULE: 18S RRNA;
411:	3mmy-G	17.0	4.0	273	355	12	PDB	MOLECULE: MRNA EXPORT FACTOR;
412:	5eap-A	17.0	3.5	253	310	10	PDB	MOLECULE: WD REPEAT-CONTAINING PROTEIN 5;
413:	5eam-A	17.0	3.4	252	304	10	PDB	MOLECULE: WD REPEAT-CONTAINING PROTEIN 5;
414:	4aez-A	17.0	3.9	262	326	12	PDB	MOLECULE: WD REPEAT-CONTAINING PROTEIN SLP1;
415:	2ymu-B	17.0	3.3	251	577	12	PDB	MOLECULE: WD-40 REPEAT PROTEIN;
416:	4ql1-B	17.0	3.5	254	302	10	PDB	MOLECULE: WD REPEAT-CONTAINING PROTEIN 5;
417:	3vgz-B	16.9	4.1	253	321	11	PDB	MOLECULE: UNCHARACTERIZED PROTEIN YNCE;
418:	4e5z-B	16.9	3.2	262	402	6	PDB	MOLECULE: DNA DAMAGE-BINDING PROTEIN 1;
419:	3mkq-E	16.9	3.5	256	810	9	PDB	MOLECULE: COATOMER BETA'-SUBUNIT;
420:	5ear-A	16.9	3.5	252	301	10	PDB	MOLECULE: WD REPEAT-CONTAINING PROTEIN 5;
421:	3vh0-D	16.8	4.2	256	322	11	PDB	MOLECULE: UNCHARACTERIZED PROTEIN YNCE;
422:	1nr0-A	16.8	3.6	258	610	10	PDB	MOLECULE: ACTIN INTERACTING PROTEIN 1;
423:	4e54-B	16.8	3.2	260	402	6	PDB	MOLECULE: DNA DAMAGE-BINDING PROTEIN 1;
424:	3mkq-C	16.8	3.5	257	811	9	PDB	MOLECULE: COATOMER BETA'-SUBUNIT;
425:	2ynp-A	16.8	3.5	256	601	9	PDB	MOLECULE: COATOMER SUBUNIT BETA';
426:	2yno-A	16.8	3.5	253	310	9	PDB	MOLECULE: COATOMER SUBUNIT BETA';
427:	2yno-B	16.8	3.6	256	310	9	PDB	MOLECULE: COATOMER SUBUNIT BETA';
428:	1vt4-I	16.7	3.0	280	1215	0	PDB	MOLECULE: APAF-1 RELATED KILLER DARK;
429:	1pev-A	16.7	3.9	270	610	6	PDB	MOLECULE: ACTIN INTERACTING PROTEIN 1;
430:	3fm0-A	16.7	4.0	266	328	9	PDB	MOLECULE: PROTEIN CIAO1;
431:	4gga-A	16.7	4.0	264	314	11	PDB	MOLECULE: CELL DIVISION CYCLE PROTEIN 20 HOMOLO
432:	5juy-C	16.7	3.8	277	1139	10	PDB	MOLECULE: APOPTOTIC PROTEASE-ACTIVATING FACTOR
433:	4ggd-A	16.7	3.9	264	312	11	PDB	MOLECULE: CELL DIVISION CYCLE PROTEIN 20 HOMOLO
434:	5juy-F	16.7	3.8	275	1139	10	PDB	MOLECULE: APOPTOTIC PROTEASE-ACTIVATING FACTOR
435:	2ymu-A	16.6	3.3	244	577	11	PDB	MOLECULE: WD-40 REPEAT PROTEIN;
436:	5juy-B	16.6	3.8	276	1234	10	PDB	MOLECULE: APOPTOTIC PROTEASE-ACTIVATING FACTOR
437:	5c2v-B	16.6	4.0	261	352	10	PDB	MOLECULE: HYDRAZINE SYNTHASE ALPHA SUBUNIT;
438:	5lcw-R	16.6	4.2	267	383	11	PDB	MOLECULE: ANAPHASE-PROMOTING COMPLEX SUBUNIT 1;
439:	5juy-E	16.6	3.8	275	1234	10	PDB	MOLECULE: APOPTOTIC PROTEASE-ACTIVATING FACTOR
440:	5juy-G	16.6	3.8	275	1234	10	PDB	MOLECULE: APOPTOTIC PROTEASE-ACTIVATING FACTOR
441:	5juy-D	16.6	3.8	275	1234	10	PDB	MOLECULE: APOPTOTIC PROTEASE-ACTIVATING FACTOR
442:	4ggd-B	16.6	3.9	263	312	11	PDB	MOLECULE: CELL DIVISION CYCLE PROTEIN 20 HOMOLO
443:	4ggc-A	16.6	4.0	264	313	11	PDB	MOLECULE: CELL DIVISION CYCLE PROTEIN 20 HOMOLO
444:	5juy-A	16.6	3.8	275	1139	10	PDB	MOLECULE: APOPTOTIC PROTEASE-ACTIVATING FACTOR
445:	5vh9-B	16.6	3.6	260	354	7	PDB	MOLECULE: DYNEIN HEAVY CHAIN, CYTOPLASMIC;
446:	4yd8-A	16.5	3.1	242	346	7	PDB	MOLECULE: PROTEIN PTHB1;
447:	5lcw-Q	16.5	4.2	267	354	11	PDB	MOLECULE: ANAPHASE-PROMOTING COMPLEX SUBUNIT 1;
448:	3ukr-C	16.5	3.8	266	349	7	PDB	MOLECULE: ACTIN-RELATED PROTEIN 3;
449:	3uku-C	16.5	3.8	265	341	7	PDB	MOLECULE: ACTIN-LIKE PROTEIN 3;
450:	1gxr-B	16.5	3.3	245	324	12	PDB	MOLECULE: TRANSDUCIN-LIKE ENHANCER PROTEIN 1;
451:	4n14-A	16.5	3.9	264	313	11	PDB	MOLECULE: CELL DIVISION CYCLE PROTEIN 20 HOMOLO
452:	3vl1-B	16.5	3.4	255	416	8	PDB	MOLECULE: 26S PROTEASOME REGULATORY SUBUNIT RPN
453:	1tyq-C	16.5	3.8	267	342	7	PDB	MOLECULE: ACTIN-RELATED PROTEIN 3;
454:	5tf2-A	16.4	3.5	246	338	11	PDB	MOLECULE: PROLACTIN REGULATORY ELEMENT-BINDING
455:	1k8k-C	16.4	3.8	264	354	7	PDB	MOLECULE: ACTIN-LIKE PROTEIN 3;
456:	5emj-B	16.4	3.9	261	303	11	PDB	MOLECULE: PROTEIN ARGININE N-METHYLTRANSFERASE
457:	4av9-A	16.4	3.8	260	320	9	PDB	MOLECULE: SVP1-LIKE PROTEIN 2;
458:	3vu4-B	16.4	3.8	257	301	11	PDB	MOLECULE: KMHSV2;
459:	5vlj-C	16.4	3.5	258	354	8	PDB	MOLECULE: DYNEIN HEAVY CHAIN, CYTOPLASMIC;
460:	2p9s-C	16.4	3.8	267	341	7	PDB	MOLECULE: ACTIN-LIKE PROTEIN 3;
461:	2p9l-C	16.4	3.9	266	341	8	PDB	MOLECULE: ACTIN-LIKE PROTEIN 3;
462:	6eoj-A	16.3	3.4	259	1232	8	PDB	MOLECULE: PROTEIN CFT1;

463:	5emk-B	16.3	4.1	266	313	11	PDB	MOLECULE: PROTEIN ARGININE N-METHYLTRANSFERASE
464:	3rse-C	16.3	3.8	266	354	7	PDB	MOLECULE: ACTIN-RELATED PROTEIN 3;
465:	3vl1-A	16.3	3.5	255	416	7	PDB	MOLECULE: 26S PROTEASOME REGULATORY SUBUNIT RPN
466:	2p9i-C	16.3	3.8	265	341	8	PDB	MOLECULE: ACTIN-LIKE PROTEIN 3;
467:	2p9p-C	16.3	3.8	265	341	7	PDB	MOLECULE: ACTIN-LIKE PROTEIN 3;
468:	2p9k-C	16.3	3.9	266	341	8	PDB	MOLECULE: ACTIN-LIKE PROTEIN 3;
469:	2p9u-C	16.3	3.9	265	341	8	PDB	MOLECULE: ACTIN-LIKE PROTEIN 3;
470:	4x63-B	16.3	4.0	266	309	12	PDB	MOLECULE: PROTEIN ARGININE N-METHYLTRANSFERASE
471:	5n1a-A	16.2	3.4	245	703	11	PDB	MOLECULE: UTP4;
472:	1jof-A	16.2	4.5	277	365	10	PDB	MOLECULE: CARBOXY-CIS,CIS-MUCONATE CYCLASE;
473:	5mps-J	16.2	3.8	259	342	7	PDB	MOLECULE: YEAST UBC4 GENE FOR UBIQUITIN-CONJUGA
474:	5g04-R	16.2	4.0	263	370	11	PDB	MOLECULE: ANAPHASE-PROMOTING COMPLEX SUBUNIT 1;
475:	4pby-A	16.2	3.8	253	384	10	PDB	MOLECULE: HISTONE-BINDING PROTEIN RBBP4;
476:	4exv-A	16.2	3.8	255	311	10	PDB	MOLECULE: SVP1-LIKE PROTEIN 2;
477:	4r7a-B	16.2	3.9	256	366	9	PDB	MOLECULE: PHD FINGER PROTEIN 6;
478:	5emm-B	16.2	4.1	268	313	11	PDB	MOLECULE: PROTEIN ARGININE N-METHYLTRANSFERASE
479:	5eml-B	16.2	4.0	263	310	10	PDB	MOLECULE: PROTEIN ARGININE N-METHYLTRANSFERASE
480:	4ui9-R	16.2	3.9	260	387	12	PDB	MOLECULE: ANAPHASE-PROMOTING COMPLEX SUBUNIT 1;
481:	4cvb-A	16.1	3.5	304	562	13	PDB	MOLECULE: ALCOHOL DEHYDROGENASE;
482:	5nuv-A	16.1	3.3	243	301	14	PDB	MOLECULE: AUTOPHAGY-RELATED PROTEIN 16-1;
483:	5n1a-B	16.1	3.3	244	706	10	PDB	MOLECULE: UTP4;
484:	4jsp-C	16.1	4.3	262	317	11	PDB	MOLECULE: SERINE/THREONINE-PROTEIN KINASE MTOR;
485:	4v16-A	16.1	4.1	260	299	10	PDB	MOLECULE: SVP1-LIKE PROTEIN 2;
486:	3jcm-B	16.1	4.2	271	428	10	PDB	MOLECULE: PRE-MRNA-SPLICING FACTOR 8;
487:	5c9z-B	16.1	4.1	266	313	11	PDB	MOLECULE: PROTEIN ARGININE N-METHYLTRANSFERASE
488:	1gxr-A	16.1	4.2	257	335	9	PDB	MOLECULE: TRANSDUCIN-LIKE ENHANCER PROTEIN 1;
489:	4x61-B	16.1	4.0	260	302	10	PDB	MOLECULE: PROTEIN ARGININE N-METHYLTRANSFERASE
490:	4yhc-A	16.0	3.8	286	439	10	PDB	MOLECULE: STEROL REGULATORY ELEMENT-BINDING PRO
491:	2co0-A	16.0	3.5	251	304	9	PDB	MOLECULE: WD-REPEAT PROTEIN 5;
492:	5tzs-U	16.0	3.8	251	284	0	PDB	MOLECULE: 5' EXTERNAL TRANSCRIBED SPACER;
493:	2p9n-C	16.0	3.9	265	341	7	PDB	MOLECULE: ACTIN-LIKE PROTEIN 3;
494:	3psl-A	16.0	3.5	246	298	9	PDB	MOLECULE: WD REPEAT-CONTAINING PROTEIN 5;
495:	1vyh-T	16.0	3.9	260	310	8	PDB	MOLECULE: PLATELET-ACTIVATING FACTOR ACETYLHYDR
496:	3iyt-A	15.9	3.3	251	1107	7	PDB	MOLECULE: APOPTOTIC PROTEASE-ACTIVATING FACTOR
497:	5o9z-F	15.9	3.3	251	420	9	PDB	MOLECULE: PRE-MRNA-PROCESSING-SPLICING FACTOR 8
498:	4ql1-A	15.9	3.5	252	304	9	PDB	MOLECULE: WD REPEAT-CONTAINING PROTEIN 5;
499:	5mps-o	15.9	3.7	248	313	7	PDB	MOLECULE: YEAST UBC4 GENE FOR UBIQUITIN-CONJUGA
500:	4j0x-B	15.8	3.6	254	366	7	PDB	MOLECULE: RIBOSOMAL RNA-PROCESSING PROTEIN 9;
501:	5m2n-A	15.8	3.7	257	711	10	PDB	MOLECULE: ELONGATOR COMPLEX PROTEIN 2;
502:	4czv-A	15.8	3.3	242	324	9	PDB	MOLECULE: PAB-DEPENDENT POLY(A)-SPECIFIC RIBONU
503:	5k0y-T	15.8	3.0	235	329	12	PDB	MOLECULE: RNA (75-MER);
504:	6eoj-D	15.8	3.5	238	388	8	PDB	MOLECULE: PROTEIN CFT1;
505:	5k1a-F	15.8	4.2	255	600	10	PDB	MOLECULE: UBIQUITIN CARBOXYL-TERMINAL HYDROLASE
506:	4v0n-D	15.8	4.1	250	304	7	PDB	MOLECULE: BARDET-BIEDL SYNDROME 1 PROTEIN;
507:	3c99-A	15.8	4.0	250	380	10	PDB	MOLECULE: CHROMATIN ASSEMBLY FACTOR 1 P55 SUBUN
508:	1pgu-A	15.8	3.9	260	606	8	PDB	MOLECULE: ACTIN INTERACTING PROTEIN 1;
509:	1vyh-S	15.8	3.9	260	310	7	PDB	MOLECULE: PLATELET-ACTIVATING FACTOR ACETYLHYDR
510:	6emk-B	15.8	3.3	261	300	8	PDB	MOLECULE: SERINE/THREONINE-PROTEIN KINASE TOR2;
511:	3acp-A	15.7	3.4	247	417	11	PDB	MOLECULE: WD REPEAT-CONTAINING PROTEIN YGL004C;
512:	3va6-A	15.7	3.5	251	747	12	PDB	MOLECULE: TWO-COMPONENT SYSTEM SENSOR HISTIDINE
513:	6bly-B	15.7	4.0	243	363	10	PDB	MOLECULE: CLEAVAGE AND POLYADENYLATION SPECIFIC
514:	6bm0-B	15.7	3.9	242	363	9	PDB	MOLECULE: CLEAVAGE AND POLYADENYLATION SPECIFIC
515:	1sq9-A	15.7	4.5	266	378	7	PDB	MOLECULE: ANTIVIRAL PROTEIN SKI8;
516:	3uvl-A	15.7	3.6	250	303	9	PDB	MOLECULE: WD REPEAT-CONTAINING PROTEIN 5;
